# Airway remodelling rather than cellular infiltration characterizes both type2 cytokine biomarker‐high and ‐low severe asthma

**DOI:** 10.1111/all.15376

**Published:** 2022-05-25

**Authors:** Latifa Khalfaoui, Fiona A. Symon, Simon Couillard, Beverley Hargadon, Rekha Chaudhuri, Steve Bicknell, Adel H. Mansur, Rahul Shrimanker, Timothy S. C. Hinks, Ian D. Pavord, Stephen J. Fowler, Vanessa Brown, Lorcan P. McGarvey, Liam G. Heaney, Cary D. Austin, Peter H. Howarth, Joseph R. Arron, David F. Choy, Peter Bradding

**Affiliations:** ^1^ Department of Respiratory Sciences, Leicester Respiratory NIHR BRC, Glenfield Hospital University of Leicester Leicester UK; ^2^ NIHR Oxford Respiratory BRC, Nuffield Department of Medicine University of Oxford Oxford UK; ^3^ Gartnavel General Hospital, Glasgow, and Institute of Infection, Immunity and Inflammation University of Glasgow Glasgow UK; ^4^ University of Birmingham and Heartlands Hospital University Hospitals Birmingham NHS Foundation Trust Birmingham UK; ^5^ School of Biological Sciences, Faculty of Biology, Medicine and Health, Manchester Academic Health Science Centre and NIHR Manchester Biomedical Research Centre, Manchester University Hospitals NHS Foundation Trust University of Manchester Manchester UK; ^6^ Wellcome‐Wolfson‐ Centre for Experimental Medicine Queen's University Belfast School of Medicine Dentistry and Biomedical Sciences Belfast UK; ^7^ Genentech, Inc. South San Francisco California USA; ^8^ School of Clinical and Experimental Sciences, NIHR Southampton Biomedical Research Centre University of Southampton Southampton UK

**Keywords:** cytokine, eosinophil, FeNO, severe asthma, Th2

## Abstract

**Background:**

The most recognizable phenotype of severe asthma comprises people who are blood eosinophil and FeNO‐high, driven by type 2 (T2) cytokine biology, which responds to targeted biological therapies. However, in many people with severe asthma, these T2 biomarkers are suppressed but poorly controlled asthma persists. The mechanisms driving asthma in the absence of T2 biology are poorly understood.

**Objectives:**

To explore airway pathology in T2 biomarker‐high and ‐low severe asthma.

**Methods:**

T2 biomarker‐high severe asthma (T2‐high, *n* = 17) was compared with biomarker‐intermediate (T2‐intermediate, *n* = 21) and biomarker‐low (T2‐low, *n* = 20) severe asthma and healthy controls (*n* = 28). Bronchoscopy samples were processed for immunohistochemistry, and sputum for cytokines, PGD_2_ and LTE_4_ measurements.

**Results:**

Tissue eosinophil, neutrophil and mast cell counts were similar across severe asthma phenotypes and not increased when compared to healthy controls. In contrast, the remodelling features of airway smooth muscle mass and MUC5AC expression were increased in all asthma groups compared with health, but similar across asthma subgroups. Submucosal glands were increased in T2‐intermediate and T2‐low asthma. In spite of similar tissue cellular inflammation, sputum IL‐4, IL‐5 and CCL26 were increased in T2‐high versus T2‐low asthma, and several further T2‐associated cytokines, PGD_2_ and LTE_4_, were increased in T2‐high and T2‐intermediate asthma compared with healthy controls.

**Conclusions:**

Eosinophilic tissue inflammation within proximal airways is suppressed in T2 biomarker‐high and T2‐low severe asthma, but inflammatory and structural cell activation is present, with sputum T2‐associated cytokines highest in T2 biomarker‐high patients. Airway remodelling persists and may be important for residual disease expression beyond eosinophilic exacerbations.

Registered at ClincialTrials.gov: NCT02883530.

## Graphical abstract

People with T2‐high and T2‐low severe asthma, and healthy controls, have similar airway tissue inflammatory cells counts, but sputum T2 cytokines persist in T2‐high asthma, likely driving eosinophilic exacerbations through recruitment of blood eosinophils. In both T2‐high and T2‐low asthma, extensive airway wall remodelling is present and likely contributes to residual disease expression beyond T2 exacerbations. As bronchoconstriction and mucus plugging are the predominant causes of airflow obstruction driving asthma symptoms, exacerbations and death, the factors that sustain these abnormal pathological features should be a priority for future research and drug development for severe asthma.Abbreviations: CCL, C‐C motif chemokine ligand; Eos, eosinophil; FeNO, fraction of exhaled nitric oxide; GC, goblet cell expressing MUC5AC; IL, interleukin; LTE4, leukotriene E4; MC, mast cell; Neu, neutrophil; T2, type2 cytokine; PGD2, prostaglandin D2; TSLP, thymic stromal lymphopoietin

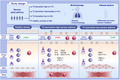

**Abbreviations**
ACQ: asthma control questionnaire
AHR: airway hyperresponsiveness
ASM: airway smooth muscle
BTS: British Thoracic Society
FEV_1_
: forced expiratory volume
GINA: Global Initiative for Asthma
HVS: Healthy Volunteer Study
ICS: inhaled corticosteroid
IQR: interquartile range
LT: leukotriene
MBP: major basic protein
MC_T_
: tryptase only mast cell
MC_TC_
: tryptase and chymase mast cell
PG: prostaglandin
RBM: reticular basement membrane
RTU: ready‐to‐use
SEM: standard error mean
T2: type 2 cytokines (IL‐4, IL‐5, IL‐13)
T2‐high‐FNS: T2 biomarker‐high FeNO non‐suppressor
T2‐intermediate: T2 biomarker‐intermediate
T2‐low: T2 biomarker‐low

## 1 INTRODUCTION

Asthma is a common, chronic and persistent disorder that accounts for significant morbidity and mortality, costing the European Union ~17.7 billion euros/annum.^1^, ^2^, ^3^ Approximately, 10% of patients have asthma which is resistant to current therapies.^2^, ^3^ This group consumes 50–60% of health care costs attributed to asthma, reflecting a considerable unmet clinical need.

Asthma is characterized by the presence of airway inflammation, airway remodelling and airway hyperresponsiveness (AHR). How these features interact and the extent of their interdependence is unclear. Furthermore, while many airway elements are implicated (epithelial dysfunction, mucous gland hyperplasia with mucus hypersecretion, airway smooth muscle [ASM] dysfunction and inflammatory cell activation), the relationships between these elements and molecular pathways are poorly understood.

Approximately, 80% of people with mild corticosteroid‐naïve asthma demonstrate evidence of a blood or airway eosinophilia, with a concomitant increase in the fraction of exhaled nitric oxide (FeNO),^4^, ^5^ although up to 95% of people with severe asthma have current or historical evidence of eosinophilia.^6^, ^7^ This eosinophilic phenotype is often characterized by increased expression of an airway gene expression signature driven by IL‐4 and IL‐13,^8^, ^9^, ^10^, ^11^ although tissue eosinophilia is also dependent on IL‐5.^12^ Together, these cytokines are described as Th2 or type 2 (T2) cytokines. Monoclonal antibodies that specifically target the IL‐4Rα inhibiting IL‐4/−13, or which target IL‐5/IL‐5Rα, demonstrate the greatest therapeutic effect in people with severe asthma who are T2 biomarker‐high (FeNO and blood eosinophils for IL‐4/−13, blood eosinophils for anti‐IL‐5).^13^, ^14^ The introduction of these drugs has transformed the treatment of people with T2‐driven severe asthma, who by definition are relatively resistant to inhaled corticosteroids (ICS).

Little is known about the molecular pathways active in severe asthma that is not T2 cytokine/biomarker‐high. These patients respond poorly to ICS and T2 cytokine‐targeted treatments, and represent a major unmet clinical need. Transcriptomic analyses from several airway biopsy studies have demonstrated mutually exclusive expression of the prototypic T2 cytokine‐dependent gene signature and an IL‐17‐dependent gene signature in mild–severe asthma.^10^, ^11^ However, 50% of uncontrolled severe asthma patients on high‐dose corticosteroid treatment are neither T2‐high nor IL‐17‐high.^10^, ^11^ This clearly represents a major challenge as it is not known what drives asthma in the absence of T2 cytokine or IL‐17 signalling. Potential mechanisms include structural change (remodelling) with fixed airflow obstruction and/or intrinsic ASM dysfunction. Ongoing mast cell activation is evident across severe asthma phenotypes,^15^ and although neutrophil‐driven disease has been proposed, neutrophil counts in the bronchial mucosa were no different between health and mild, moderate or severe asthma in many studies.^16^, ^17^, ^18^ Some studies have also suggested there is over activity of interferon‐γ^19^, ^20^, ^21^ which might aggravate AHR.^19^

The aim of this study was to explore the airway immunopathology of people at the extremes of the T2 biomarker spectrum in order to define the key features that contribute to corticosteroid insensitive T2‐high severe asthma and T2‐low severe asthma.

## 2 METHODS

Detailed methods are available in the Appendix S1.

### 2.1 Ethics and consent

The RASP bronchoscopy study was a multi‐centre study, which recruited people with severe asthma prospectively using pre‐defined inclusion and exclusion criteria. It was approved by the East Midlands—Leicester South Research Ethics Committee (REC) (reference 16/EM/0260) and registered at ClinicalTrials.gov (NCT02883530). Biopsy samples collected using the same standard operating procedure were also used from the pre‐intervention arms of two other studies, i) a single‐centre bronchoscopy study assessing the effects of ICS on adult healthy volunteers (referred to as Leicester HVS from here) (NCT02476825, REC approval 15/EM/0313) (only pre‐intervention baseline biopsies were studied), and ii) a multi‐centre bronchoscopy study evaluating the effects of lebrikizumab on airway eosinophilic inflammation in uncontrolled asthma^22^ (referred to as Genentech CLAVIER from here) (NCT02099656, independent ethics committee approval was obtained at all participating centres; only pre‐intervention baseline biopsies were studied). All participants gave written informed consent. The Leicester HVS ran contemporaneously with RASP and used the same standard operating procedure and the same bronchoscopist for both studies (PB).

### 2.2 Study population

Detailed inclusion and exclusion criteria are provided in the online repository text. People with asthma aged 18–70 were eligible, and all were current non‐smokers with a < 15 pack‐year smoking history.

Standard criteria for the diagnosis of asthma are summarized in the Appendix S1. Patients were deemed adherent in their asthma centre through the prior analysis of prescription refills, measurement of prednisolone and cortisol levels if appropriate, and/or FeNO suppression testing.^23^, ^24^ Current use of a biologic treatment was an exclusion criteria, and only one patient had used a biologic previously (omalizumab).

Participants underwent extensive evaluation at baseline including a full medical history, lung function testing, bronchial challenge using methacholine where appropriate, and induced sputum.

People with severe asthma were recruited prospectively and identified as follows: (i) patients with a previous FeNO ≥45 ppb and blood eosinophils ≥0.3 × 10^9^/L, who did not suppress their FeNO during a FeNO suppression test^23^, ^24^, ^25^ during routine clinical care, referred to as T2 biomarker‐high FeNO non‐suppressors (T2‐high‐FNS); (ii) patients with a FeNO ≤30 ppb and blood eosinophils ≤0.2 × 10^9^/L identified in clinic, referred to as T2 biomarker‐low (T2‐low); this group was supplemented with 9 participants from the Genentech CLAVIER study.^22^ (iii) patients who had exited the RASP T2‐biomarker (FeNO, blood eosinophils, periostin)‐driven treatment optimization study^6^ with either intermediate biomarker measurements, referred to as T2 biomarker‐intermediate (T2‐intermediate) or low T2 biomarkers (as described for ii above). The rationale for the biomarker levels defining the T2‐low group is provided in the Appendix S1.

Healthy volunteers in Leicester HVS had no prior history or clinical evidence of lower respiratory disease and normal spirometry. Healthy volunteers with a history of rhinitis (perennial or seasonal) were required to have a PC_20_ methacholine >16 mg/ml.

### 2.3 Standard operating procedures

All centres were experienced in performing research bronchoscopies in asthma. RASP, Leicester HVS and CLAVIER used the same standard operating procedures for tissue collection and processing, and all immunohistochemical staining and analysis were performed in Leicester. These are summarized briefly as follows:

### 2.4 Bronchoscopy

Subjects underwent bronchoscopy conducted according to British Thoracic Society guidelines.^26^ Mucosal biopsies were collected from 2nd to 5th generation bronchi under direct vision as per study procedure manual.

### 2.5 Immunohistochemistry

Biopsies were fixed in 4% neutral buffered formalin for 4 h at 4°C as described for the Clavier study,^22^ then processed into paraffin wax, as per study procedure manual, with the same protocol used for all studies contributing to this analysis. Immunohistochemistry was performed in Leicester. All the laboratory procedures and processes were performed following the ISO9001‐2015 Quality Management System and GCP/GLP guidelines. Further immunostaining details are provided in the Appendix S1.

### 2.6 Assessment of immunopathology

High‐throughput morphologic analysis was performed on scanned sections using a Carl Zeiss Scanner Z1 and AxioCam HRc digital camera (Carl Zeiss, Germany) and ZEN desk 3.1 image analysis software. The following previously validated pathological features^17^ were evaluated in whole sections as follows: (i) nucleated inflammatory cells (eosinophils, neutrophils, mast cells [tryptase+, chymase+]) in the airway epithelium, lamina propria, ASM bundles and airway glands, expressed as cells/mm;^2^ (ii) the area occupied by epithelial, ASM and glandular structures expressed as a percentage of the total biopsy area; (iii) MUC5AC expressed as the percentage of airway epithelium staining positive;^16^ (iv) reticular basement membrane (RBM) thickness expressed in microns.^27^ The mean of two sections at least 18 μm apart was taken for each immunohistological analysis.

All pathological data were assessed by an observer blinded to the identity of the patient.

### 2.7 Sputum mediators

Induced sputum supernatants were collected in PBS. Cytokines (IL‐4/−5/−13/−31/−33, CCL17/26, TARC, TSLP, IFNγ and TNFα), prostaglandin (PG)D_2_ and leukotriene (LT)E_4_ were measured as described in the Appendix S1.

### 2.8 Statistical analysis

The approach to statistical analysis is provided in the Appendix S1.

## 3 RESULTS

### 3.1 Clinical characteristics

For bronchoscopy, we recruited 54 patients with asthma prospectively, with suitable tissue for immunohistochemical analysis available from 49, with an additional 9 T2‐low patients from the CLAVIER study and 28 healthy controls from the Leicester HVS. The clinical characteristics of the pre‐defined bronchoscopy study groups are presented in Table 1.

**Table 1:** Baseline demographic data for the bronchoscopy cohort

	T2‐high FeNO‐NS [*n* = 17]	T2‐intermediate [*n* = 21]	T2‐low [*n* = 20]	Healthy (*n* = 28)	*p* value†
Age—years	57 (50–63)**	56 (46.5–63)**	51 (35.3–58.3)	27 (22–50)	**<0.0001**
Sex—M/F	9/8	11/10	13/7	14/14	0.7596
BMI (kg/m^2^)	29.8 (24.9–32.6)**	31.4 (26.8–38.7)****	30.3 (28.2–33.8)***	23.8 (21.4–27.3)	**<0.0001**
Age onset—years	28.8 ± 19.2	29.6 ± 19.7	18.4 ± 14.0	N/A	0.0935
Ethnicity Caucasian—%	100	90.5	89.5	78.6	0.1647
Asthma duration—years	27.2 ± 19.6	25.4 ± 14.8	29.4 ± 12.7	N/A	0.7166
Atopic^a^—%	86.7	61.1	82.4	32.1	**0.0007**
Severe annual exacerbation frequency	2.0 (1.0–3.5)	1.0 (0.0–2.0)	1.0 (1.0–5.0)	N/A	0.1188
ICS dose—BDP equivalent—mcg	2000 (2000–2000)	2000 (1000–2400)	2000 (2000–2000)	N/A	0.4632
Maintenance oral corticosteroids—%	29.4	33.3	20.0	N/A	0.6213
Oral corticosteroid dose—mg	10.0 (5–20) [*n* = 5]	10.0 (5–20) [*n* = 7]	8.75 (5–10) [*n* = 4]	N/A	0.7618
Long‐acting β2‐agonist—%	100	100	100	N/A	1.0000
Long‐acting muscarinic antagonist—%	52.9	38.1	45.0	N/A	0.6578
Leukotriene receptor antagonist—%	47.1	52.4	15.0	N/A	**0.0313**
Theophylline—%	41.2	14.3	5.0	N/A	**0.0158**
Ex smoker—%	29.4	33.3	35.0	14.3	0.3285
Smoking—pack‐years	0 (0.0–2.0)	0.0 (0.0–4.5)	0.0 (0.0–0.9)	0.0 (0.0–0.0)	0.3153
FEV_1_ Pre‐BD—L	2.02 (1.66–2.71)****	2.43 (2.02–2.79)***	2.55 (2.02–3.15) **	3.59 (2.89–4.08)	**<0.0001**
FEV_1_ Pre‐BD—% predicted	66.8 (55.1–80.7)****	78.3 (72.4–94.0)**	71.4 (63.2–88.0)****	102.0 (94.5–109.8)	**<0.0001**
FEV_1_/FVC—%	59.4 (55.2–66.4)****	70.5 (62.6–78.3)**	64.9 (56.1–73.5)****	81.0 (77.3–84.9)	**<0.0001**
ACQ5	1.6 (0.8–2.3)	1.2 (0.6–1.9)	2.1 (1.7–3.1)¶	N/A	**0.0352**
Total IgE—kU/L	132 (94–274)	69 (17–338)	173 (73.8–350.3)	Not done	0.4372
FeNO—ppb	71.0 (40.0–104.5) ****/ ##/§§§§	21 (15.0–31.0)	15.0 (11.3–19.5)	17.5 (12.3–25.3)	**<0.0001**
Blood eosinophils (screening)—×10^9^/L	0.38 (0.23–0.58)****/ #/§§§§	0.17 (0.08–0.31)	0.11 (0.07–0.17)	0.09 (0.06–0.14)	**<0.0001**
Blood eosinophils (highest recorded)—×10^9^/L	0.81 (0.60–1.12)#	0.5 (0.24–0.66)	0.45 (0.31–0.70) (*n* = 11)	Not available	**0.0228**
Sputum eosinophils—%	10.7 (0.8–35.3) §§ [*n* = 8]	1.2 (0.0–6.7) [*n* = 14]	0.0 (0.0–0.3) [*n* = 15]	Not done	**0.0067**
Sputum neutrophils—%	23.5 ± 27.9 [*n* = 8]	47.4 ± 36.7 [*n* = 14]	49.9 ± 27.7 [*n* = 15]	Not done	**0.1068**

Continuous variables are presented as mean ± SD or median (interquartile range). BMI = Body Mass Index. BD = Bronchodilator. †All tests for continuous variables are ANOVA or Kruskal–Wallis across all groups unless indicated otherwise, with adjusted *p*‐values for between‐group comparisons obtained using Sidak's or Dunn's multiple comparison tests. For categorical variables, a Chi‐squared test was used across applicable groups.

*p < 0.05, **p < 0.01, ***p < 0.001, ****p < 0.0001 compared with healthy control subjects. #*p* < 0.05, ##*p* < 0.01 compared with T2‐intermediate. §§§*p* < 0.001, §§§§*p* < 0.0001 compared with T2‐low. ¶*p* < 0.05 compared with T2‐intermediate.

^a^ Atopy refers to the presence of a positive skin test or the presence of a raised specific IgE to a common aeroallergen.

Both FeNO and blood eosinophils were markedly increased in the T2‐high‐FNS compared with the other groups, as expected from the entry requirements for this group. This phenotype, therefore, appears stable over time (Table 1). Sputum eosinophils were also highest in T2‐high‐FNS, but T2‐low patients were the most symptomatic (Table 1). Many T2‐intermediate and T2‐low patients had historical evidence of raised blood eosinophils (Table 1).

### 3.2 Tissue inflammatory cells

All subjects had lamina propria suitable for analysis of inflammatory cells, but there were fewer with suitable epithelium, ASM and submucosal glands for inflammatory cell analysis within these compartments, as shown in the corresponding figures. Immunostaining from one centre was unsuccessful for major basic protein and neutrophil elastase (*n* = 7 subjects), and these markers were excluded from the analysis.

**FIGURE 1:**
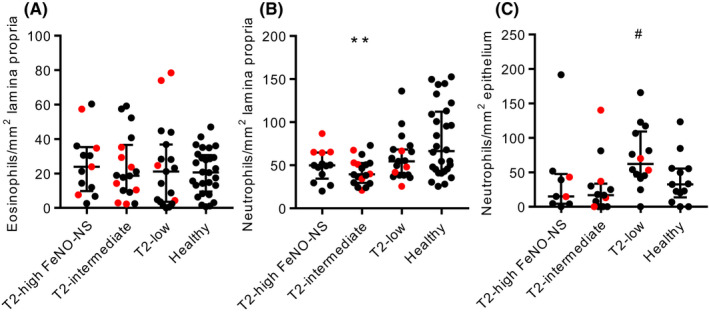
Eosinophil and neutrophil numbers in asthma subgroups and healthy controls. A) Eosinophil numbers in the airway lamina propria (Kruskal–Wallis *p* = 0.8444). B) Neutrophil numbers in the airway lamina propria (Kruskal–Wallis *p* = 0.0119). C) Neutrophil numbers in the airway epithelium (Kruskal–Wallis: *p* = 0.0197 all groups, *p* = 0.0105 asthma groups). **p = 0.0043 compared with healthy controls, #*p* = 0.0120 compared with T2‐intermediate (Dunn's multiple comparison test). Red points represent people taking oral corticosteroids

Representative immunostaining is shown in Figure S1. Eosinophil counts in the airway lamina propria were similar in asthma and health (Figure S2A) and across asthma groups (Figure 1A). Eosinophils were rarely seen in the airway epithelium (Figure S2B,C), only present in the airway submucosal gland stroma in 3 asthmatics, and never seen within ASM.

Neutrophil counts in the lamina propria were lower in asthma compared with health (*p* = 0.0052) (Figure S2D), with no differences between the asthma subgroups (Figure 1B). This difference between asthma and health was lost when the healthy control group was matched to those with asthma for age by removing people under the age of 25 (age did not influence any other immunohistological parameters—see online repository for further details). Neutrophil counts in the airway epithelium were similar between asthma and health (Figure S2E), but there was a significant difference across the asthma subgroups (Kruskal–Wallis *p* = 0.0105) (Figure 1C), with more epithelial neutrophils in T2‐low compared with T2‐intermediate asthma (*p* = 0.0120). Neutrophils were present within the ASM bundles in 2 asthmatics and 2 healthy controls and the airway gland stroma of 3 asthmatics.

**FIGURE 2:**
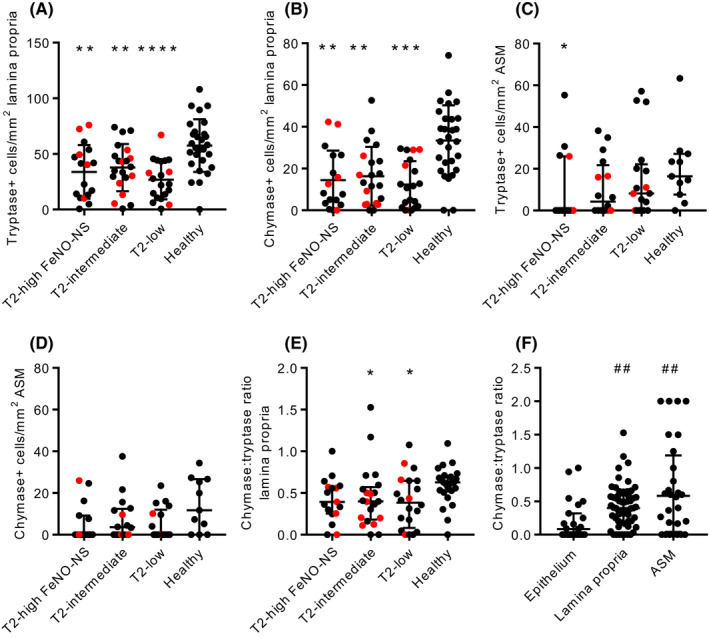
Mast cell numbers in asthma subgroups and healthy controls. A) Tryptase+ mast cell numbers in the airway lamina propria (ANOVA *p* < 0.0001). B) Chymase+ mast cell numbers in the airway lamina propria (Kruskal–Wallis *p* < 0.0001). C) Tryptase+ mast cell numbers within the airway smooth muscle (ASM) (Kruskal Wallis *p* = 0.0923). D) Chymase+ mast cell numbers within the ASM (Kruskal–Wallis *p* = 0.1346). E) The chymase:tryptase ratio in the lamina propria (Kruskal–Wallis *p* = 0.0280). F) The chymase:tryptase ratio in the airway epithelium, lamina propria and ASM in people with asthma (Kruskal–Wallis, *p* = 0.0018) (where chymase+ cells were present but there were no tryptase+ cells, values of 2 were given). For A‐E) *p < 0.05, **p < 0.01, ***p < 0.001, ****p < 0.0001 compared with healthy controls (Dunnett's or Dunn's multiple comparison tests as appropriate). For F) ##*p* < 0.01 compared with airway epithelium (Dunn's multiple comparison test). Red points represent people taking oral corticosteroids

Tryptase+ (total) and chymase+ mast cell counts were reduced in the airway lamina propria in severe asthma compared with health in keeping with previous studies^18^ (*p* < 0.0001) (Figure S3A and B) and similar in the asthma subgroups (Figure 2A and B). Tryptase+ mast cell counts in the airway epithelium were similar in asthma and health (Figure S3C) and similar in the asthma subgroups (Figure S3D). Chymase+ mast cells were also present in the airway epithelium but in much lower numbers than tryptase+ mast cells (Figure S3E and F).

Tryptase+ mast cells were present in the airway glands in 9/19 asthmatics who had glandular tissue present and 2/3 healthy controls. These cells rarely contained chymase, with chymase present in only 3 patients. Tryptase+ and chymase+ mast cells were often present within the ASM with reduced chymase+ numbers in asthma compared with health (*p* = 0.0514 and *p* = 0.0263, respectively) (Figure S3G and H). There were no differences between the asthma groups (Figure 2C and D).

The ratio of chymase+:tryptase+ mast cells was significantly lower in asthmatic lamina propria compared with healthy lamina propria (*p* = 0.0022) (Figure S3I), but similar across asthma subgroups (Figure 2E), and demonstrated a gradient from the airway epithelium, through the lamina propria to the ASM in asthma (Kruskal–Wallis *p* = 0.0018) (Figure 2F).

### 3.3 Tissue remodelling

**FIGURE 3:**
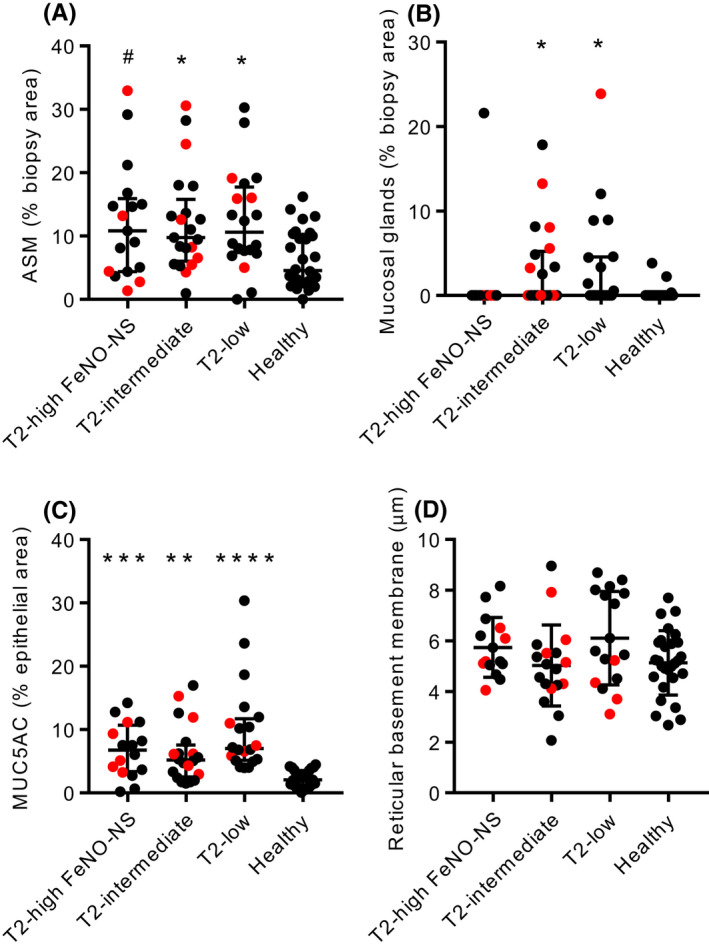
Remodelling features in asthma subgroups and healthy controls. A) Airway smooth muscle (ASM) mass expressed as a percentage of total biopsy area (Kruskal–Wallis *p* = 0.0098). B) Mucosal gland mass expressed as a percentage of total biopsy area (Kruskal–Wallis *p* = 0.0026). C) MUC5AC immunostaining expressed as a percentage of epithelial area (Kruskal–Wallis *p* < 0.0001). D) Reticular basement membrane (RBM) thickness (ANOVA *p* = 0.0910). *p < 0.05, **p < 0.01, ***p < 0.001, ****p < 0.0001 compared with healthy controls (Dunn's multiple comparison test). For A), #*p* = 0.0506 compared with healthy (Dunn's multiple comparison test). Red points represent people taking oral corticosteroids

Airway smooth muscle area expressed as a percentage of total biopsy area was increased in asthma versus health (*p* = 0.0006) (Figure S4) and to a similar extent across the different asthma subgroups (Figure 3A). Airway submucosal glands expressed as a percentage of total biopsy area were also increased in asthma versus health (*p* = 0.0150) (Figure S4), accounted for by increases in the T2‐intermediate and T2‐low asthma groups (Figure 3B). There was a significant difference across the asthma subgroups in submucosal glandular tissue area (Kruskal–Wallis *p* = 0.0386), but no significant post hoc difference between asthma subgroups. MUC5AC expressed as the percentage of airway epithelium staining was elevated in asthma compared with healthy controls (*p* < 0.0001) (Figure S4), with similar increases in all asthma subgroups (Figure 3C).

There were no differences between asthma and health or across the asthma groups for epithelial area expressed as percentage of airway biopsy total area (Figure S4), or for reticular basement membrane thickness (Figure 3D and Figure S4).

Post hoc re‐analysis of the data restricting the T2‐low group to blood eosinophils ≤0.15x109/L and FeNO ≤25 ppb produced similar results for both inflammatory cell infiltration and remodelling features (Figure S9).

### 3.4 Sputum cytokines, PGD_2_ , LTE_4_

Sputum supernatants were available from 34/54 of the bronchoscopy cohort described above, 2 people without bronchoscopic samples collected for immunohistochemistry, and 7 participants who passed screening but did not proceed to bronchoscopy, commonly because they withdrew or exacerbated. The clinical characteristics of these 43 patients and 6 healthy controls are summarized in Appendix S2 and are similar to the primary bronchoscopy cohort. A subset of these subjects have been described previously.^28^

**FIGURE 4:**
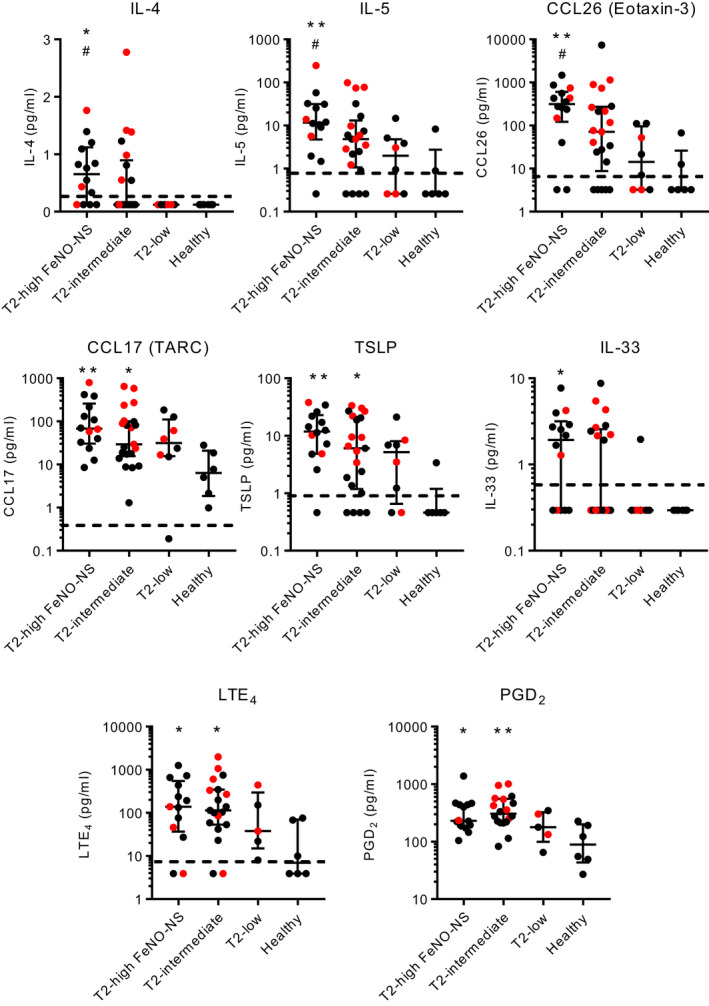
T2‐associated cytokine/chemokine, LTE_4_ and PGD_2_ concentrations in induced sputum supernatants in asthma subgroups and healthy controls. *p < 0.05, **p < 0.01, compared with healthy controls (Dunn's multiple comparison test). #*p* < 0.05 compared with T2‐low (Dunn's multiple comparison test). Red points represent people taking oral corticosteroids. Dotted lines represent the lower limit of detection

IL‐5, CCL17, CCL26 and TSLP concentrations were elevated significantly in asthma overall compared with health (Figure S5). IL‐4, IL‐5 and CCL26 were significantly higher in T2‐high‐FNS compared with both T2‐low asthma and healthy controls (Figure 4). CCL17 and TSLP concentrations were increased in T2‐high‐FNS and T2‐intermediate asthma compared with healthy controls, but similar across the asthma groups (Figure 4). IL‐33 was increased in T2‐high‐FNS compared with healthy controls (Figure 4). There were no differences for IFNγ, IL‐31 or TNFα evident (Figure S5). Within the asthma patients (*n* = 43), there were strong positive correlations between all T2‐associated cytokines (Figure S6).

FeNO correlated with all T2 cytokines and most strongly with IL‐4 (*r*_s_ = 0.661, *p* < 0.0001). Sputum eosinophils correlated with all T2 cytokines except IL‐13 and most strongly with IL‐5 (*r*_s_ = 0.644, *p* < 0.0001). Blood eosinophils correlated weakly with most sputum T2 cytokines. FeNO correlated with blood eosinophils (*r*_s_ = 0.589, *p* < 0.0001) and sputum eosinophils (*r*_s_ = 0.487, *p* = 0.003), but the correlation between sputum and blood eosinophils was weak (*r*_s_ = 0.331, *p* = 0.045, *n* = 37) (Figure S6).

For PGD_2_ and LTE_4_, samples were available for only 5 T2‐low patients. Both PGD_2_ and LTE_4_ were elevated in asthma compared with healthy controls (Figure S7) and elevated in both T2‐high‐FNS and T2‐intermediate patients compared with health (Figure 4). Levels of PGD_2_ in T2‐low asthma were similar to those in T2‐high‐FNS, and there were no significant differences between the asthma groups (Figure 4).

## 4 DISCUSSION

We have compared the immunopathology of highly polarized severe asthma phenotypes, namely people who have persistently raised T2‐biomarkers despite electronically monitored high‐dose ICS treatment (T2‐high‐FNS), people who have had their treatment titrated against T2 biomarkers (T2‐intermediate), and people with low T2 biomarkers (T2‐low). Our aim was to explore the airway immunopathological factors that might be contributing to disease expression in these T2‐high versus T2‐low cohorts.

These asthma groups were clearly separated by their expression of two widely accepted biomarkers of T2 activity, FeNO and blood eosinophils, in spite of taking similar doses of ICS and oral corticosteroids. Both the T2‐intermediate and T2‐low groups had significantly higher blood eosinophil counts in the past, suggesting that persistent T2‐biomarker‐low asthma is rare as described.^6^, ^7^ Nevertheless, these T2‐low patients remain very symptomatic and prone to exacerbations (Table 1).

The definition for T2‐low we used was planned before the results of more recent phase 3 trials of biologic therapies became available. T2 activity is not all‐or‐nothing, but graded, and there is debate about what thresholds define T2‐low. The UK severe asthma registry now defines T2‐low as blood eosinophils <0.15 × 10^9^/L and FeNO ≤25 ppb. However, while a blood eosinophil count of (0.15 × 10^9^/L) is the quoted cut for response to IL‐5‐targeted therapies, the clinical response just above this is minimal and of doubtful clinical significance,^29^ as reflected in the UK National Institute for Clinical Excellence (NICE) prescribing criteria where the cut for treatment eligibility is 0.30 × 10^9^/L. Furthermore, the upper 95% CI for blood eosinophils in healthy controls is at least 0.2 × 10^9^/L.^30^ In our T2‐low cohort, only one patient had a FeNO >25 ppb, and that was 27 ppb, while 75% of patients had blood eos ≤0.15. The T2‐low blood eosinophil counts and FeNO levels were similar to the healthy control group. However, a post hoc analysis of the data limiting the T2‐low group to those with blood eosinophils <0.15 × 10^9^/L and FeNO ≤25 ppb does not alter the conclusions of this study, and it is clear from the sputum data that this T2‐low group had suppressed T2 cytokine expression in the airways and that our original thresholds were reasonable.

In keeping with raised FeNO and blood eosinophils in T2‐high‐FNS, there were increased concentrations of many T2‐associated cytokines in their induced sputum supernatant compared with T2‐low patients and healthy controls. These were strongly inter‐correlated and correlated with FeNO and sputum eosinophils, indicating a rich T2‐environment. However, tissue eosinophil numbers were not elevated in any of the asthma groups compared with healthy controls and were similar across the asthma groups, despite raised sputum eosinophils in the T2‐high‐FNS. These data may appear counterintuitive, but are consistent with the U‐BIOPRED cohort where there was no evidence of tissue eosinophilic inflammation in severe asthma compared with health, despite increased sputum eosinophils in severe asthma overall.^18^ Several other^16^, ^31^ but not all^17^, ^32^ studies of severe asthma concur that there is no tissue eosinophilia. This is unlikely to be because bronchoscopic sampling is insensitive as studies of mild steroid‐naïve asthma show a consistent increase in tissue eosinophils (epithelium and lamina propria), and this is reduced consistently by inhaled corticosteroids.^33^, ^34^ The discrepancy with sputum eosinophil counts in T2‐high patients suggests there is ongoing eosinophil trafficking, although we might have expected to see increased eosinophils in the airway epithelium if that is the case. Nevertheless, the high levels of eosinophil chemoattractants in sputum support this explanation, and thus, a heightened level of trafficking may explain their relatively reduced numbers in the tissue. In addition, it is possible that the survival of tissue‐resident eosinophils remains highly sensitive to the effects of ICS in stable‐state severe asthma, while production of epithelial chemokines and T2 cytokines by mast cells, ILC2 cells, Th2 T cells, and perhaps eosinophils that remain activated,^35^ are relatively resistant to the effects of ICS in T2 biomarker‐high patients.

IL‐5 and anti‐IL‐4/13‐targeted therapies are most effective in severe asthmatics expressing high type 2 biomarkers,^14^, ^36^ and IL‐5‐targeted therapy likely works exclusively through its effects on eosinophil biology. These biologic therapies reduce the rate of severe asthma exacerbations to a greater extent than improving day‐to‐day symptoms and lung function.^36^, ^37^ As tissue eosinophil numbers were not increased in stable‐state T2‐high severe asthma, the efficacy of anti‐IL5/IL‐5Rα and anti‐IL‐4Rα therapy on exacerbations may be due to their effects on systemic rather than tissue eosinophils, preventing recruitment and further trafficking to the tissue during exacerbations. This interpretation is supported by the observations that blood eosinophils are a better predictor of response to mepolizumab than sputum eosinophils,^29^, ^38^ although not all studies have shown this.^39^ However, activation of the remaining tissue eosinophils may also contribute to exacerbations.^35^ Furthermore, as IL‐4 and IL‐13 have biological effects that extend well beyond an effect on eosinophils, it is likely that anti‐IL‐4Rα therapy impacts other components of IL‐4/13 biology in asthmatic airways that promote exacerbations, for example, by attenuating the effects of IL‐4/13 on AHR.^40^, ^41^

There are significant residual exacerbations in T2‐high severe asthma treated with T2 biologics, many of which are non‐eosinophilic in origin.^42^ This, coupled with persisting symptomatology and airway dysfunction in both T2‐high and T2‐low asthma, requires explanation and represents a major unmet clinical need. Bronchoconstriction and AHR are defining features of asthma. Our study is cross‐sectional, but the presence of increased ASM mass within the airway wall across all severe asthma phenotypes when compared to healthy controls, despite intense treatment, provides further evidence that the ASM remains a key dysfunctional element driving severe asthma pathophysiology,^43^ irrespective of the underlying inflammatory milieu. The ongoing production of potent bronchoconstrictors (LTE_4_ and PGD_2_) in the airways in both T2‐high and T2‐low asthma despite high‐dose ICS+/−oral corticosteroid therapy is likely important in the presence of this hyperresponsive pathologically remodelled ASM. It also suggests that everyone with severe asthma should receive a trial of a leukotriene receptor antagonist.

Mucus secretion is another major contributor to airflow obstruction in both mild and severe asthma,^44^, ^45^, ^46^, ^47^, ^48^ particularly during exacerbations and in asthma deaths.^46^, ^49^, ^50^ MUC5AC expression was increased in the airway epithelium and to a similar extent across the asthma cohorts. Many T2 and non‐T2 cytokines, growth factors and lipid mediators potentially increase MUC5AC expression and mucus secretion.^51^, ^52^, ^53^ While T2 cytokines may make an important contribution to ongoing mucus production in T2‐high asthma and provide another mechanism through which anti‐IL4Rα therapy may work to reduce asthma exacerbations, further work is required to establish the many possible factors driving persistent MUC5AC expression in T2‐low asthma. In addition, the presence of submucosal glands was increased in asthma, although it is unclear why this signal was driven by T2‐low and T2‐intermediate asthma rather than T2‐high asthma.

Mast cells are activated by most stimuli considered important for the pathogenesis of asthma, its propagation and the development of asthma exacerbations.^54^ Their vast array of mediators can account for many of the described pathological features of asthma and the disordered airway physiology.^54^ Numerous cross‐sectional studies demonstrate that mast cells are continuously activated in asthmatic airways, irrespective of disease severity or phenotype.^15^, ^54^, ^55^ The presence of similarly high sputum PGD_2_ concentrations across the asthma groups in our study is consistent with these previous studies. Mast cell numbers in the lamina propria are similar in mild steroid‐naïve asthma compared with health^16^, ^17^, ^56^ and reduced by ICS.^57^ Some studies found similar mast cell numbers in the lamina propria in severe asthma compared with health and others reduced numbers.^16^, ^18^, ^31^ Our data are in keeping with the latter. Epithelial mast cells are increased in mild steroid‐naive asthma compared with healthy controls,^16^, ^56^ but reduced by ICS,^57^ consistent with this and previous studies.^16^, ^18^ A role for mast cells in both T2‐high and T2‐low severe asthma is supported by a recent phase 3 trial of masitinib, which reduced the rate of severe asthma exacerbations in both T2 biomarker‐high and T2 biomarker‐low severe asthma.^58^, ^59^ Taken together, this suggests that it is the activity of mast cells rather than their numbers, which is important.

Mast cell infiltration of the ASM is a characteristic feature of mild asthma^54^, ^60^ and likely important physiologically as this places activated mast cells within the ASM bundles.^61^ In our previous study investigating pathological heterogeneity, mast cell counts in the ASM were only elevated compared with healthy controls in mild–moderate asthma, but not in severe asthma.^17^ U‐BIOPRED also found no difference in the number of mast cells in the ASM in severe asthma compared with healthy controls.^18^ Our study is, therefore, in keeping with previous studies. However, mast cells within the ASM bundles in severe asthma may remain activated with important physiological consequences. Electron microscopy to assess the level of piecemeal degranulation as a feature of activation, as shown in milder asthma,^62^, ^63^ would help answer this question.

The term ‘neutrophilic asthma’ is often used to describe patients with high sputum neutrophil percentage counts without consideration of total cell counts. A sputum neutrophilia is common in people using ICS, as corticosteroids induce eosinophil apoptosis but prolong neutrophil survival, and an inverse correlation between sputum eosinophil and neutrophil counts is described.^64^ A small proportion of severe asthmatics have a high total sputum neutrophil count and often complain of repeated episodes of purulent sputum. These patients can perhaps be accurately described as having a neutrophilic phenotype.^65^ However, this and many previous studies have failed to find increased airway neutrophil infiltration in mild, moderate or severe asthma when compared to healthy controls.^16^, ^17^, ^18^, ^31^, ^66^ That is not to say that neutrophils do not play a role, because like mast cells, their activation status may be more important than absolute numbers. Increased numbers of neutrophils expressing CEACAM6 in severe asthma suggest that neutrophil activation status may indeed be altered.^66^

Our study has some limitations as it is cross‐sectional, and patients can only be bronchoscoped when clinically stable and not exacerbating. However, the remarkably similar pathology between T2‐high and T2‐low severe asthma suggests this does not fluctuate markedly over time. It is also not feasible to biopsy the distal small airways, and it is possible that the T2‐biomarker signals in breath and sputum arise from the distal airways where ICS may not reach.^67^ However, the persisting T2 signals in people on oral corticosteroids argue against this. Furthermore, T2‐dependent gene signatures in proximal bronchial biopsies were associated with sputum eosinophilia and raised FeNO levels in previous studies.^10^ Although there was no tissue inflammatory cell signal that distinguished T2‐high from T2‐low asthma, we believe that bronchoscopy as a research tool still has an important role to play in the further understanding of the pathology and molecular pathways contributing to steroid insensitivity and ongoing disease expression, exemplified by recent transcriptomic studies.^55^, ^68^ It will also continue to have an important role in understanding the efficacy on novel therapies on remodelling and inflammation and for demonstrating drug target engagement within the airways.

In summary, tissue eosinophilic inflammation is absent in both T2 biomarker‐high and T2 biomarker‐low severe asthma. Persisting T2 cytokine expression in people with T2 biomarker‐high severe asthma likely underpins the ability of T2‐targeted treatments to reduce eosinophilic asthma exacerbations, most likely by preventing the recruitment of eosinophils at the time of exacerbation. However, there are many clinical aspects of severe asthma that appear orthogonal to T2 inflammation (non‐eosinophilic exacerbations, impaired lung function and day‐to‐day symptoms), likely accounted for by persisting increases in ASM mass, glandular hyperplasia, enhanced mucus production and mast cell activation. Considering that bronchoconstriction and mucus plugging are the predominant causes of airflow obstruction driving asthma symptoms, exacerbations and death, the factors that sustain these abnormal pathological features remain an important area for future research and drug development.

## CONFLICT OF INTEREST

SC has received non‐restricted research grants from the NIHR Oxford BRC, Sanofi‐Genzyme and the Quebec Respiratory Health Research Network; he is the holder of the Association Pulmonaire du Québec's Research Chair in Respiratory medicine; he received speaker honoraria from AstraZeneca, GlaxoSmithKline, Sanofi‐Regeneron and Valeo Pharma; he received consultancy fees for FirstThought; he has received sponsorship to attend international scientific meetings by AstraZeneca. He is an advisory board member for Biometry Inc—a company which is developing a FeNO device (myBiometry); the contract will be remunerated by stock options. RC has received lecture fees from GSK, AstraZeneca, Teva, Chiesi and Sanofi; honoraria for Advisory Board Meetings from GSK, AstraZeneca, Teva, Chiesi and Novarti; sponsorship to attend international scientific meetings from Chiesi, Napp, Sanofi, Boehringer, GSK and AstraZeneca and a research grant to her Institute from AstraZeneca for a UK multi‐centre study. AHM has received personal and institutional payment for talks, advisory board meetings, education and research funding from GSK, AstraZeneca, Teva, Chiesi, NAPP, Sanofi, Novartis, PI. TSCH has received grants from Pfizer Inc., the University of Oxford, the Wellcome Trust, The Guardians of the Beit Fellowship, the NIHR Oxford Biomedical Research Centre, Sensyne Health and Kymab during the conduct of the study; and personal fees from AstraZeneca, TEVA and Peer Voice outside the submitted work. IDP, In the last 5 years, IDP has received speaker's honoraria for speaking at sponsored meetings from Astra Zeneca, Boehringer Ingelheim, Aerocrine, Almirall, Novartis, Teva, Chiesi, Sanofi/Regeneron, Menarini and GSK, and payments for organizing educational events from AstraZeneca, GSK, Sanofi/Regeneron and Teva. He has received honoraria for attending advisory panels with Genentech, Sanofi/Regeneron, Astra Zeneca, Boehringer Ingelheim, GSK, Novartis, Teva, Merck, Circassia, Chiesi and Knopp, and payments to support FDA approval meetings from GSK. He has received sponsorship to attend international scientific meetings from Boehringer Ingelheim, GSK, AstraZeneca, Teva and Chiesi. He has received a grant from Chiesi to support a phase 2 clinical trial in Oxford. In 2014–5 and 2019–20, he was an expert witness for a patent dispute involving AstraZeneca and Teva. SJF has received grants from Boehringer Ingelheim and fees from AstraZeneca, Boehringer Ingelheim, Novartis, Teva and Chiesi. LPM declares Research funding from Chiesi and Merck; consultancies for Chiesi, Glaxo Smith Kline, Merck, Sanofi, Genentech and support to attend scientific meetings from Chiesi, Merck and Bayer. LGH reports grants from Genentech/Hoffman la Roche, during the conduct of the study; other from AstraZeneca, Boehringer Ingelheim, Chiesi, GSK and Napp Pharmaceuticals, personal fees from Novartis, Hoffman la Roche/Genentech Inc, Sanofi, Evelo Biosciences, Glaxo Smith Kline, Astra Zeneca, Teva, Theravance, Circassia, grants from Medimmune, Novartis UK, Roche/Genentech Inc and Glaxo Smith Kline, Amgen, Genentech/Hoffman la Roche, Astra Zeneca, Medimmune, Glaxo Smith Kline, Aerocrine and Vitalograph, outside the submitted work. CDA is an employee of Genentech. PHH is an employee of GSK. JRA is an employee of Genentech. DC is an employee of Genentech. PB has received research funding from Genentech via the University Hospitals of Leicester NHS Trust; consultancies for Boehringer Ingelheim, Genentech and Celldex Therapeutics via the University of Leicester. Support to attend scientific meetings from Chiesi, Teva and Sanofi‐Genzyme. BH, LC, FAS, SB, RS, VB no conflict of interest.

## AUTHOR CONTRIBUTIONS

All authors reviewed the data and contributed to its interpretation, edited the manuscript and approved the final submitted version. LC and FAS: Performed immunohistochemistry and quantified and collated the data. SC: Performed cytokine measurements on induced sputum supernatants and quantified and collated the data. BH: Managed the study, contributed to patient recruitment and collated the demographic data. RC: Principal investigator in Glasgow. SB: Performed bronchoscopy in Glasgow. AHM: Principal investigator in Birmingham. RS: Recruited and bronchoscoped patients in Oxford. TSCH: Supervised sputum cytokine analysis. IDP: Principal investigator in Oxford. SJF: Principal investigator in Manchester, performed bronchoscopy. VB: Sample management and storage. LPM: Co‐Investigator in Belfast performed bronchoscopies in Belfast, on study progress committee. LGH: Principal investigator in Belfast, conceived and designed the study, academic lead for RASP‐UK programme, bronchoscopy study progress committee. CDA: Contributed to bronchoscopy protocol design and provided tissue samples from the CLAVIER cohort. PHH: Principal investigator in Southampton. JRA: Contributed to study design and provided tissue samples from the CLAVIER cohort. DC: Contributed to study design and manuscript drafting. PB: Conceived and designed the study, wrote the study protocol and ethical application, analyzed the data, drafted the manuscript and performed all bronchoscopies in Leicester. Chief investigator.

## Supporting information


Figure S1

Figure S2

Figure S3

Figure S4

Figure S5

Figure S6

Figure S7

Figure S8

Figure S9

Appendix S1

Appendix S2
