# Supplementary material for: Airway remodelling rather than cellular infiltration characterizes both type2 cytokine biomarker‐high and ‐low severe asthma
Source: Allergy. 2022 May 25;77(10):2974–86. doi: 10.1111/all.15376 (PMC9790286; doi:10.1111/all.15376)

Supplementary figure E1

Representative immunostaining

<u>T2-high FeNO non suppressor</u>

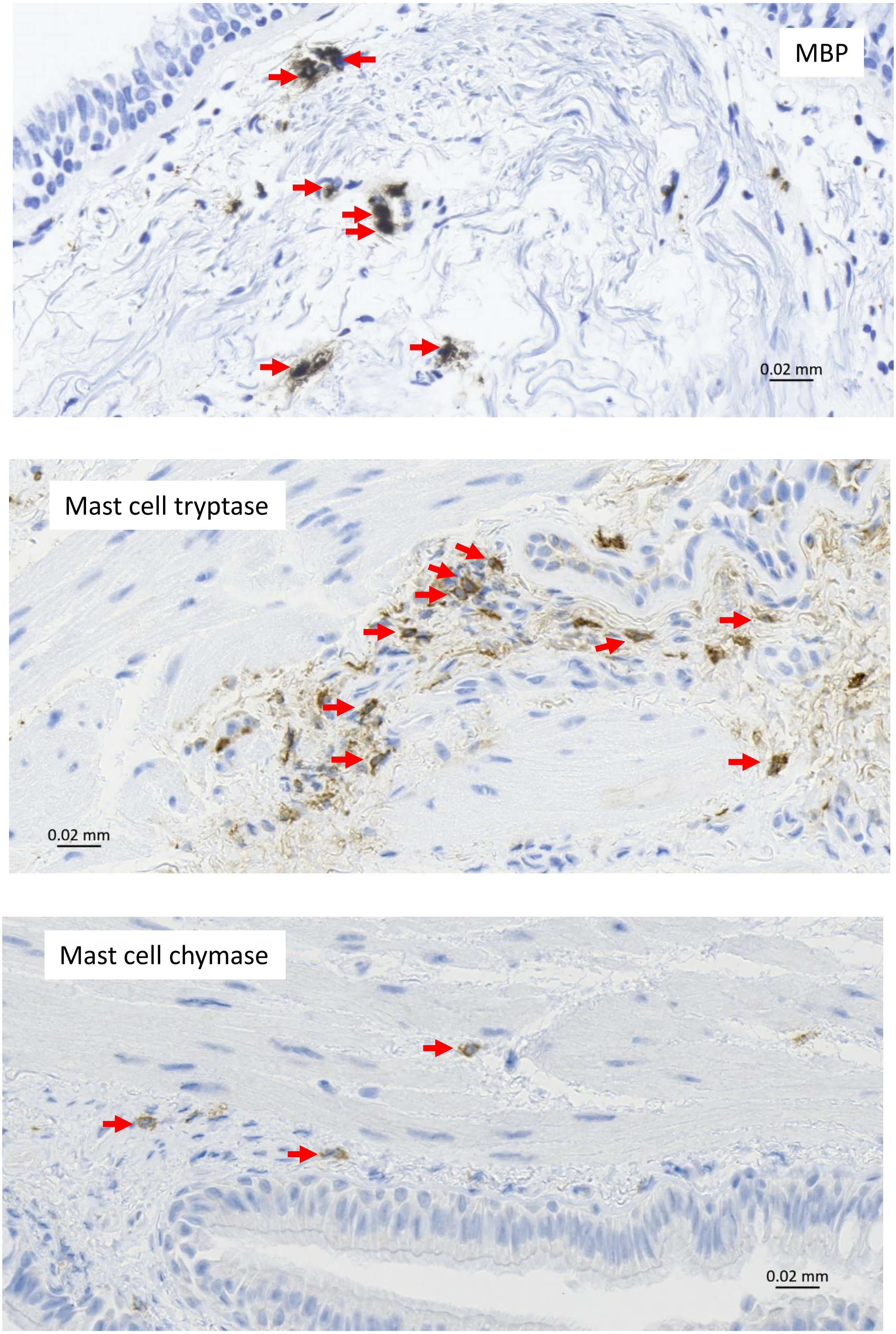

T2-high FeNO non suppressor

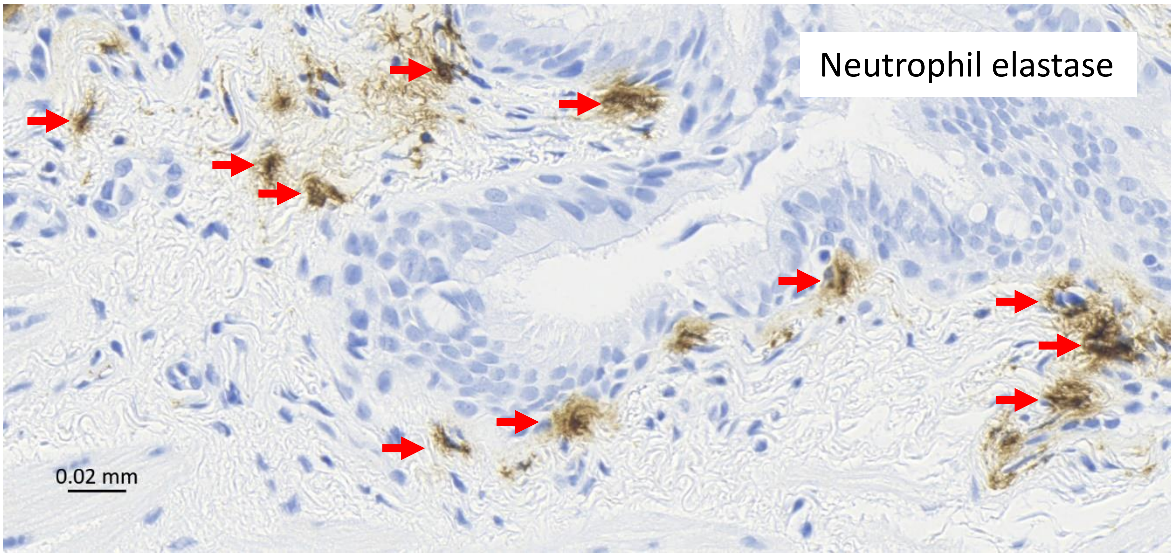

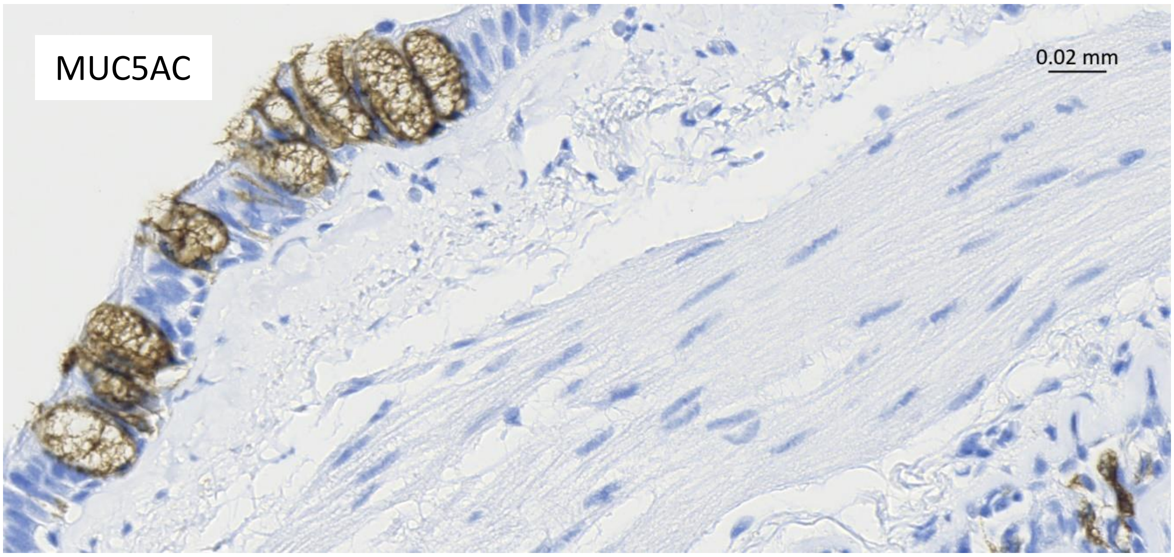

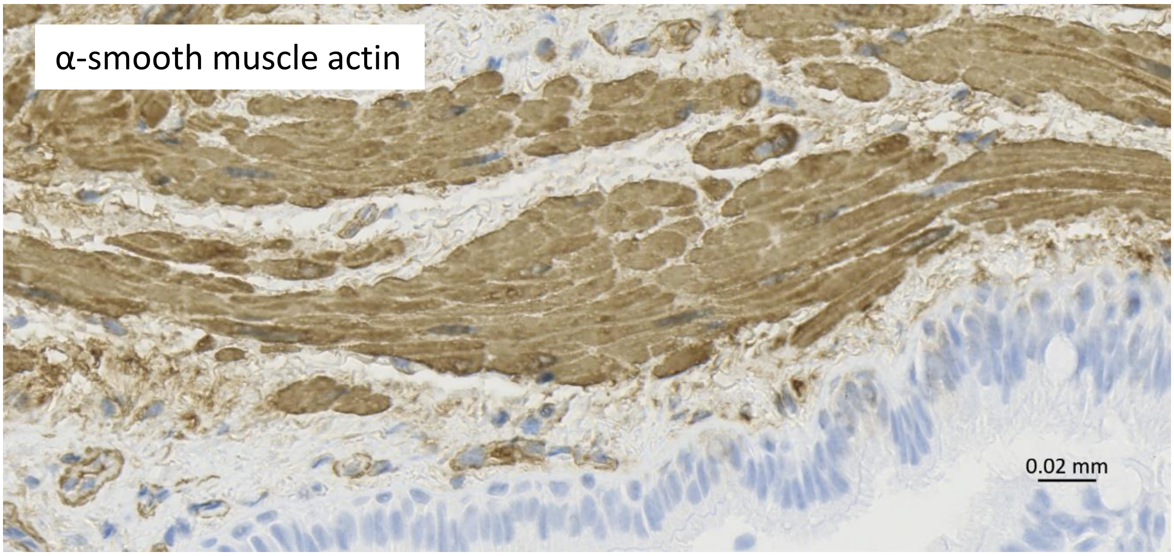

T2-low

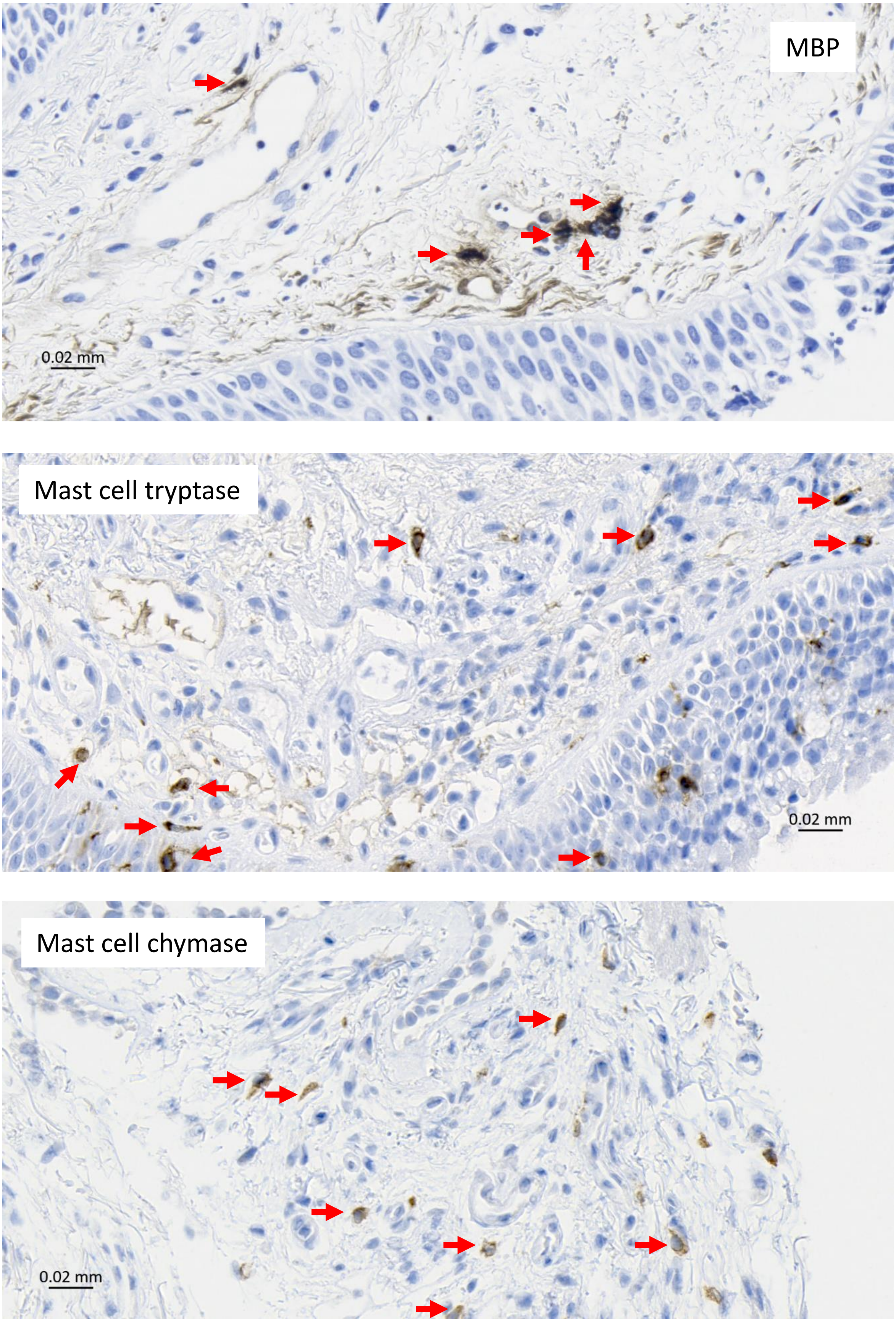

T2-low

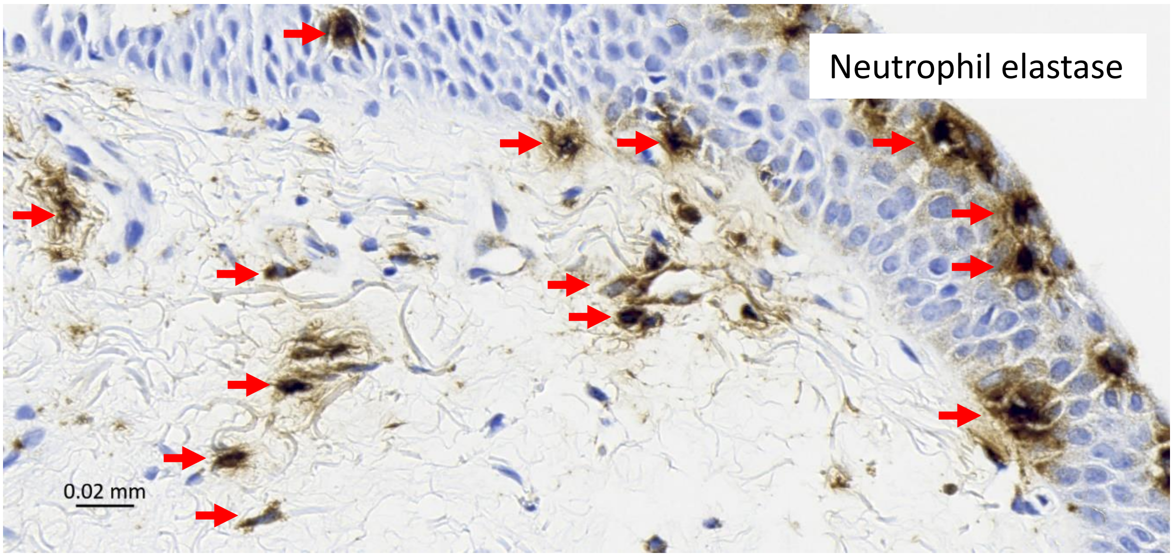
Neutrophil elastase
0.02 mm

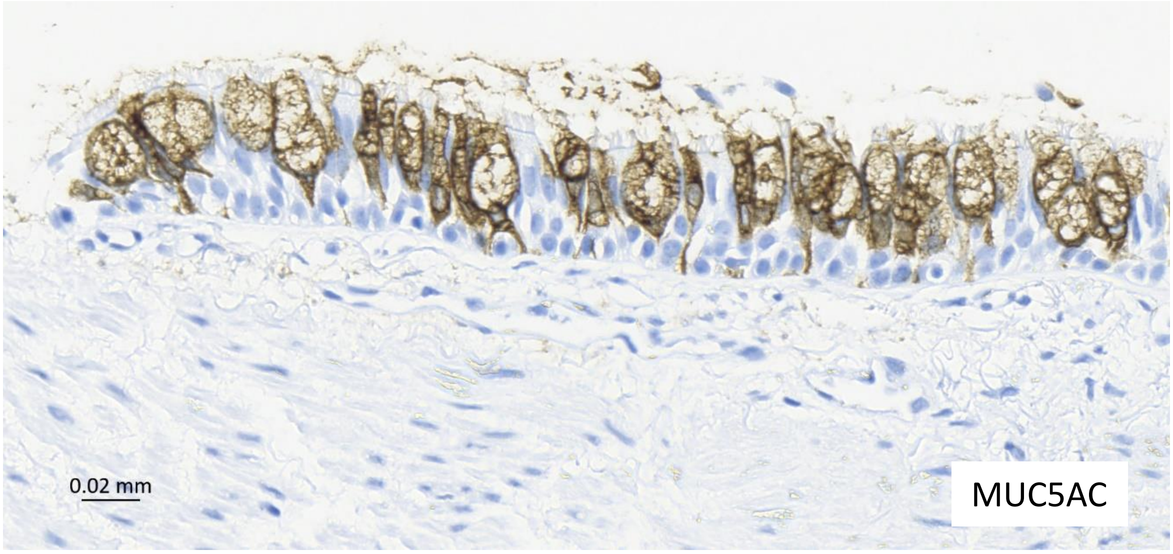
0.02 mm
MUC5AC

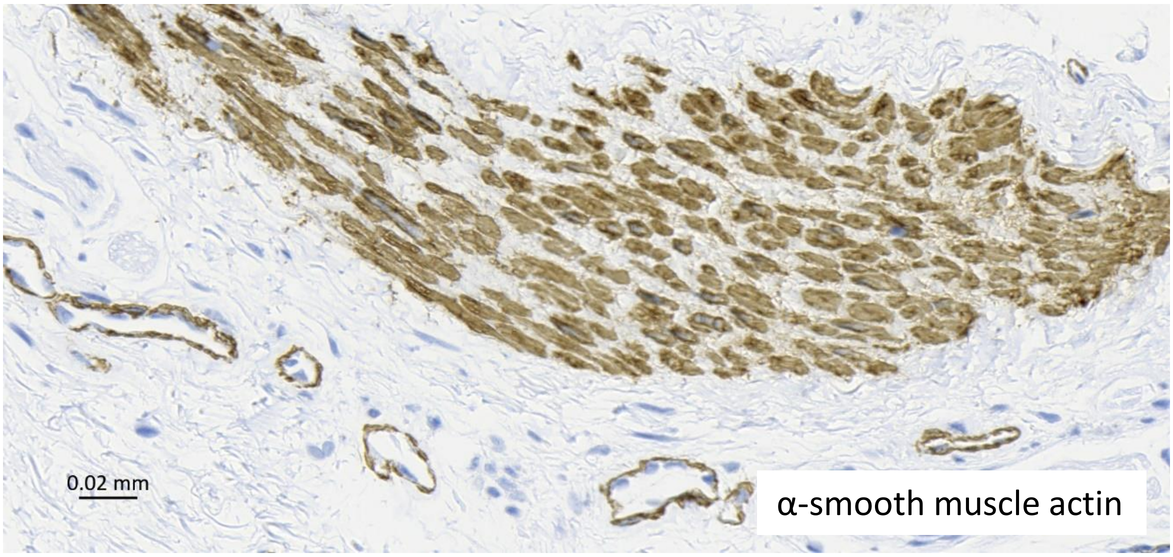
0.02 mm
α-smooth muscle actin

Healthy control

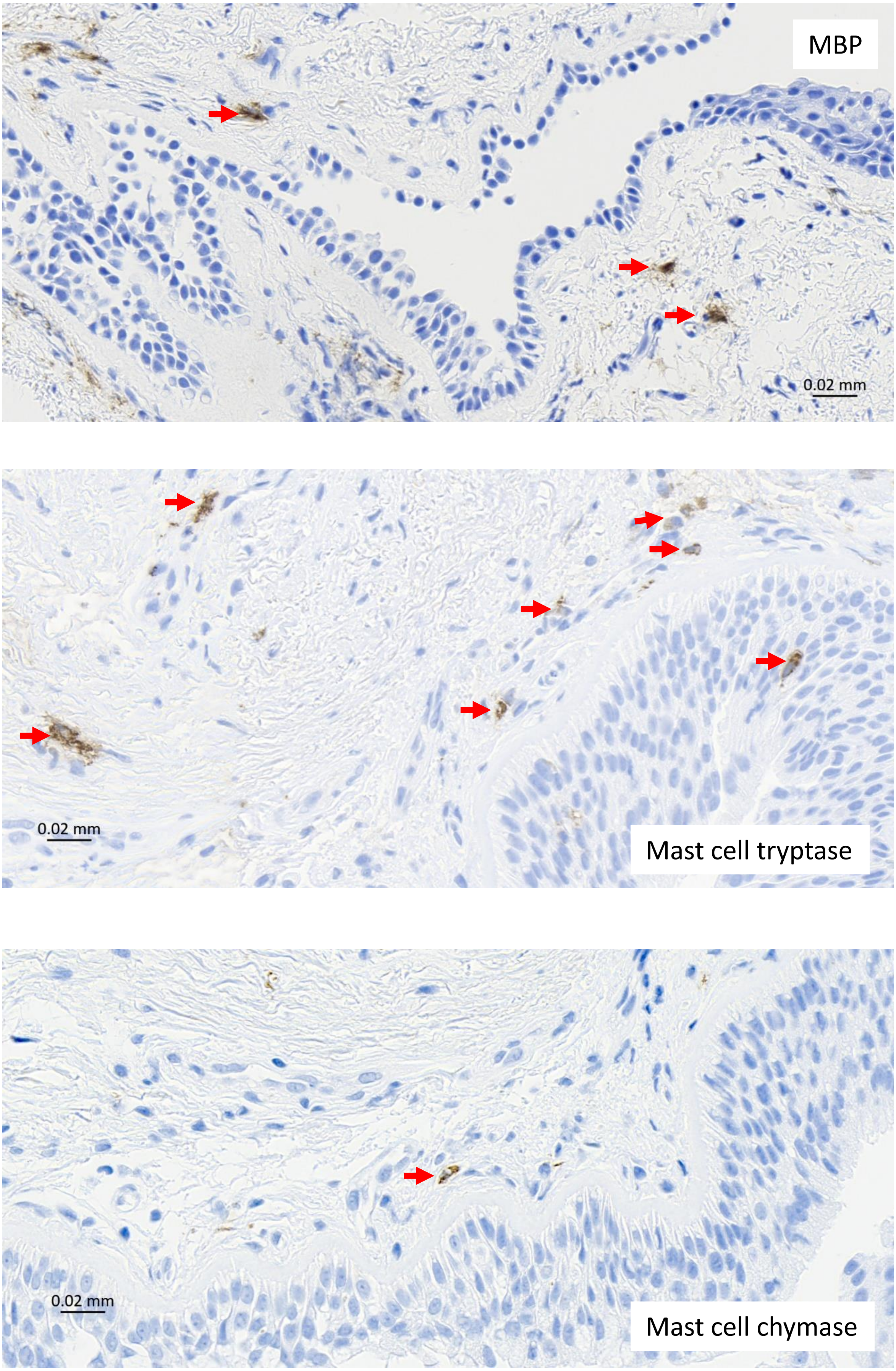

Healthy control

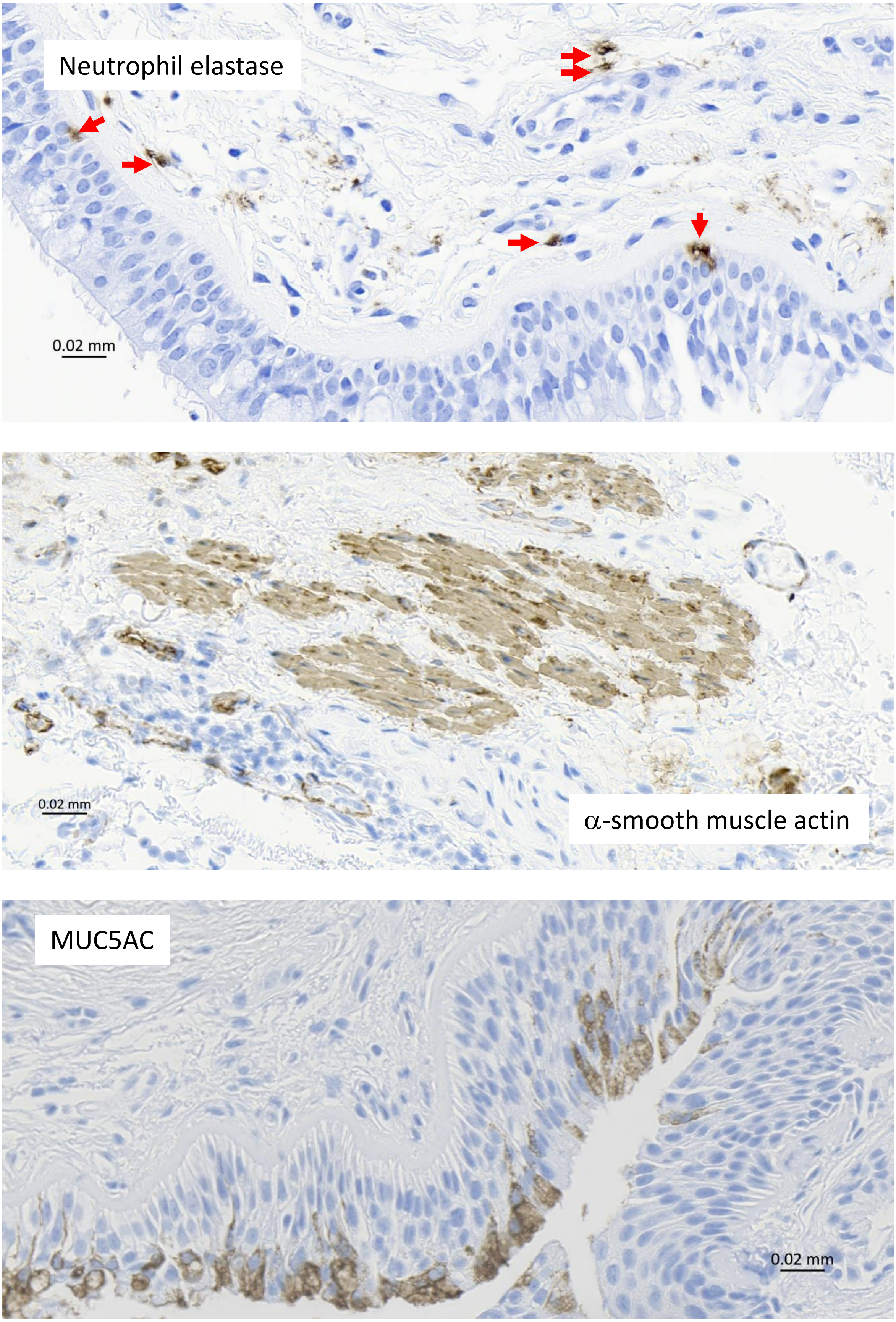

Isotype controls

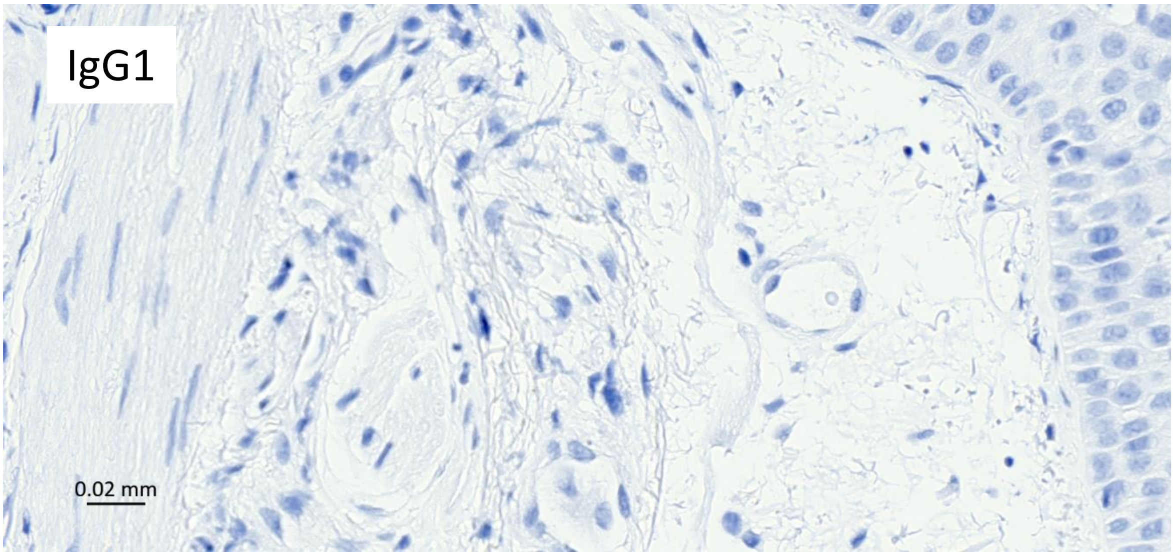

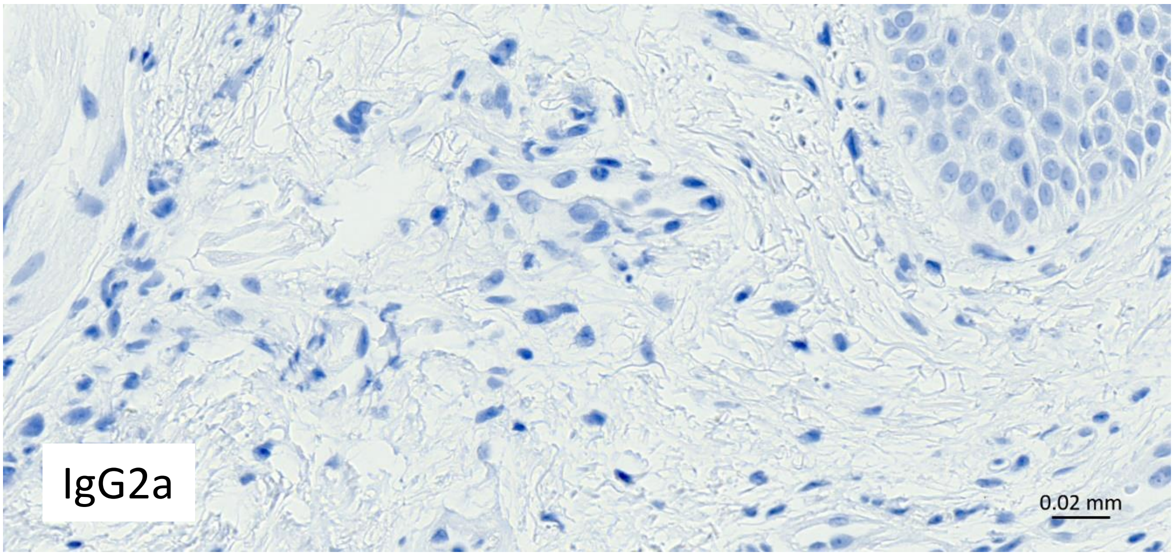

Supplement: Supplementary file 1 — Figure S1 [file ALL-77-2974-s011.pdf]
