# Supplementary figures and images for: Airway remodelling rather than cellular infiltration characterizes both type2 cytokine biomarker‐high and ‐low severe asthma
Source: Allergy. 2022 May 25;77(10):2974–86. doi: 10.1111/all.15376 (PMC9790286; doi:10.1111/all.15376)

Supplementary figure E2

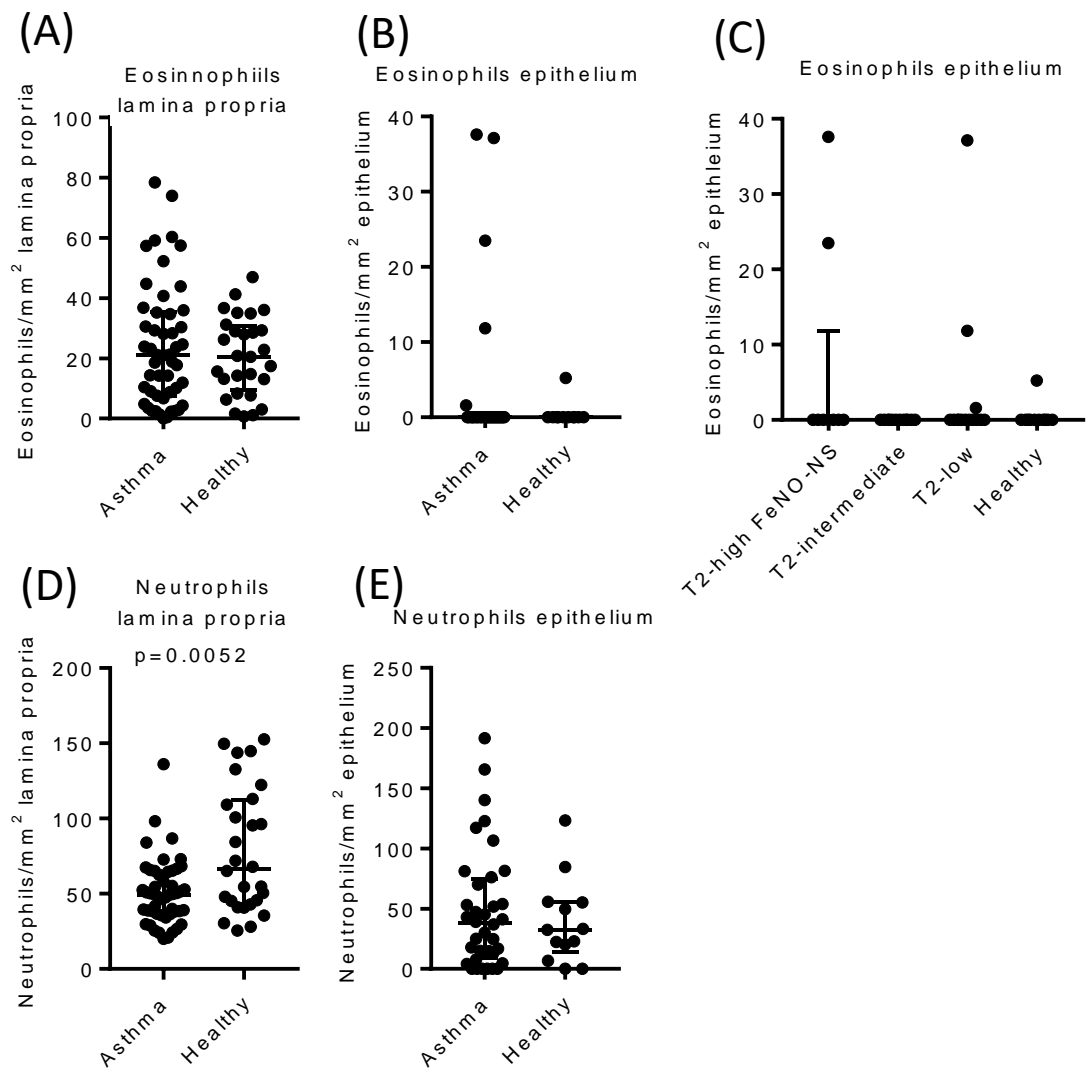

Figure E2\_Khalifaoui et al

Supplement: Supplementary file 2 — Figure S2 [file ALL-77-2974-s002.pdf]

# Supplementary figure E3

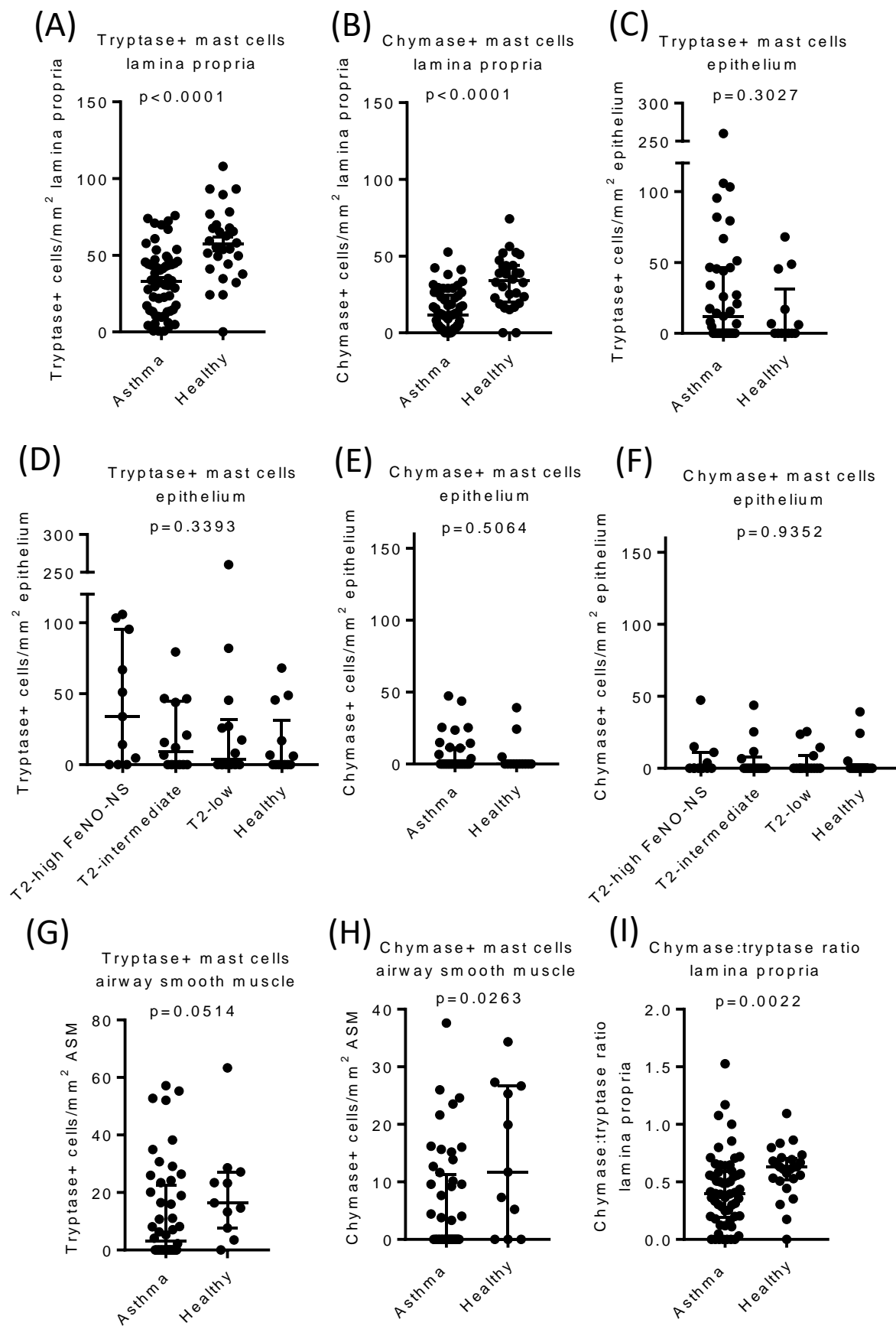

Figure E3\_Khalfaoui et al

Supplement: Supplementary file 3 — Figure S3 [file ALL-77-2974-s009.pdf]

Supplementary figure E4

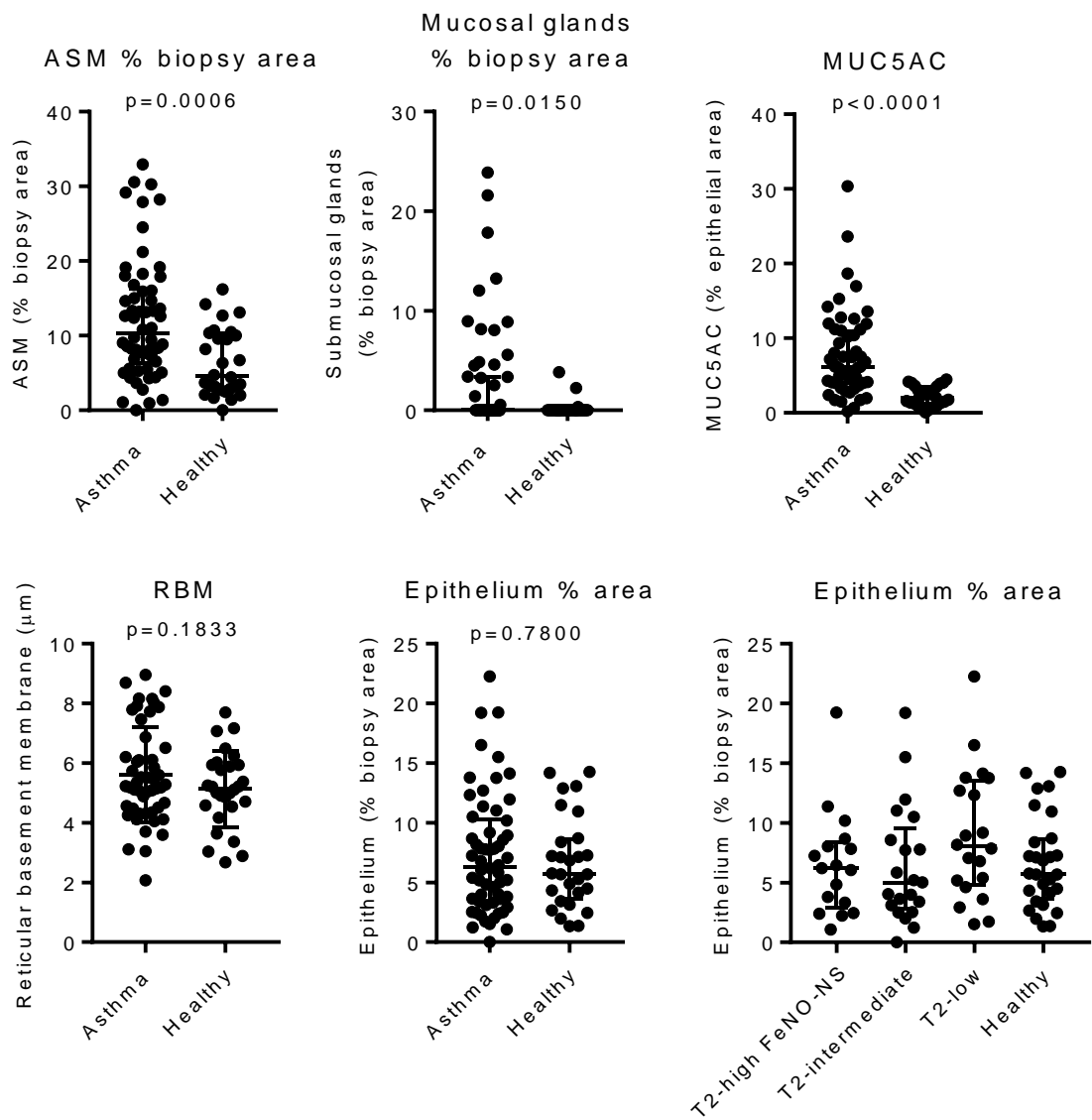

Figure E4\_Khalifaoui et al

Supplement: Supplementary file 4 — Figure S4 [file ALL-77-2974-s007.pdf]

# Supplementary figure E5

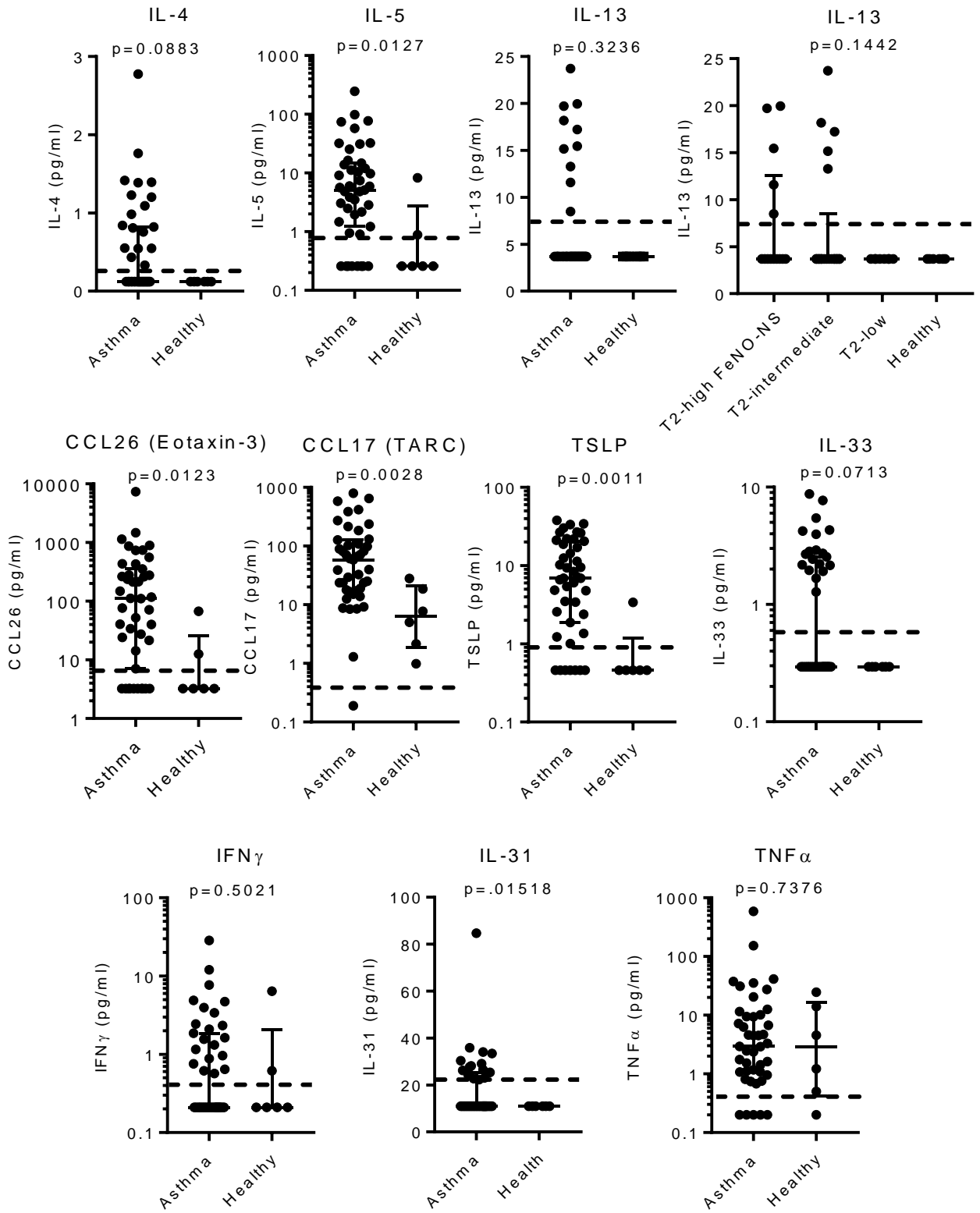

Figure E5\_Khalifaoui et al

Supplement: Supplementary file 5 — Figure S5 [file ALL-77-2974-s006.pdf]

Supplementary figure E7

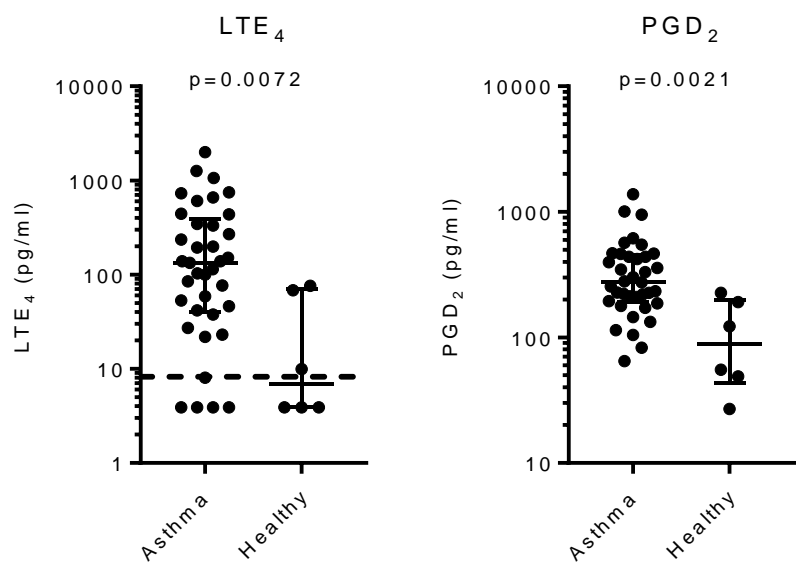

Figure E7\_Khalifaoui et al

Supplement: Supplementary file 7 — Figure S7 [file ALL-77-2974-s008.pdf]
