# Supplementary material for: Airway remodelling rather than cellular infiltration characterizes both type2 cytokine biomarker‐high and ‐low severe asthma
Source: Allergy. 2022 May 25;77(10):2974–86. doi: 10.1111/all.15376 (PMC9790286; doi:10.1111/all.15376)
Supplement: Supplementary file 6 — Figure S6 [file ALL-77-2974-s005.pdf]

# Supplementary figure E6

| | IL-13 | IL-4 | IL-5 | TSLP | CCL26 | IL-33 | CCL17 | IFNγ | FeNO | Blood eos | Sputum eos |
|---|---|---|---|---|---|---|---|---|---|---|---|
| IL-13 | | **<0.0001** | **<0.0001** | **<0.0001** | **<0.0001** | **0.0003** | **<0.0001** | **0.0005** | **0.0023** | **0.0172** | 0.0986 |
| IL-4 | 0.655 | | **<0.0001** | **<0.0001** | **<0.0001** | **0.0010** | **<0.0001** | **<0.0001** | **<0.0001** | **0.0193** | **0.0001** |
| IL-5 | 0.621 | 0.854 | | **<0.0001** | **<0.0001** | **<0.0001** | **<0.0001** | **<0.0001** | **<0.0001** | **0.0346** | **<0.0001** |
| TSLP | 0.592 | 0.705 | 0.826 | | **<0.0001** | **0.0002** | **<0.0001** | **<0.0001** | **0.0074** | 0.2602 | **0.0050** |
| CCL26 | 0.610 | 0.870 | 0.903 | 0.809 | | **0.0012** | **<0.0001** | **<0.0001** | **<0.0001** | **0.0334** | **0.0002** |
| IL-33 | 0.604 | 0.900 | 0.763 | 0.633 | 0.806 | | **0.0020** | **0.0009** | 0.2566 | 0.8462 | 0.0794 |
| CCL17 | 0.505 | 0.643 | 0.821 | 0.860 | 0.759 | 0.564 | | **<0.0001** | **<0.0001** | **0.0255** | **0.0037** |
| IFNγ | 0.522 | 0.484 | 0.609 | 0.540 | 0.478 | 0.458 | 0.487 | | **0.0457** | 0.4335 | **0.0017** |
| FeNO | 0.458 | 0.669 | 0.611 | 0.407 | 0.626 | 0.585 | 0.310 | 0.179 | | **<0.0001** | **0.0026** |
| Blood eos | 0.361 | 0.356 | 0.323 | 0.176 | 0.325 | 0.340 | 0.123 | -0.030 | 0.583 | | **0.0452** |
| Sputum eos | 0.276 | 0.595 | 0.644 | 0.452 | 0.577 | 0.465 | 0.498 | 0.292 | 0.487 | 0.331 | |

Figure E6_Khalfaoui et al
