# Supplementary material for: Airway remodelling rather than cellular infiltration characterizes both type2 cytokine biomarker‐high and ‐low severe asthma
Source: Allergy. 2022 May 25;77(10):2974–86. doi: 10.1111/all.15376 (PMC9790286; doi:10.1111/all.15376)
Supplement: Supplementary file 8 — Figure S8 [file ALL-77-2974-s004.pdf]

Supplementary figure E8
Post hoc analysis with healthy controls matched for age

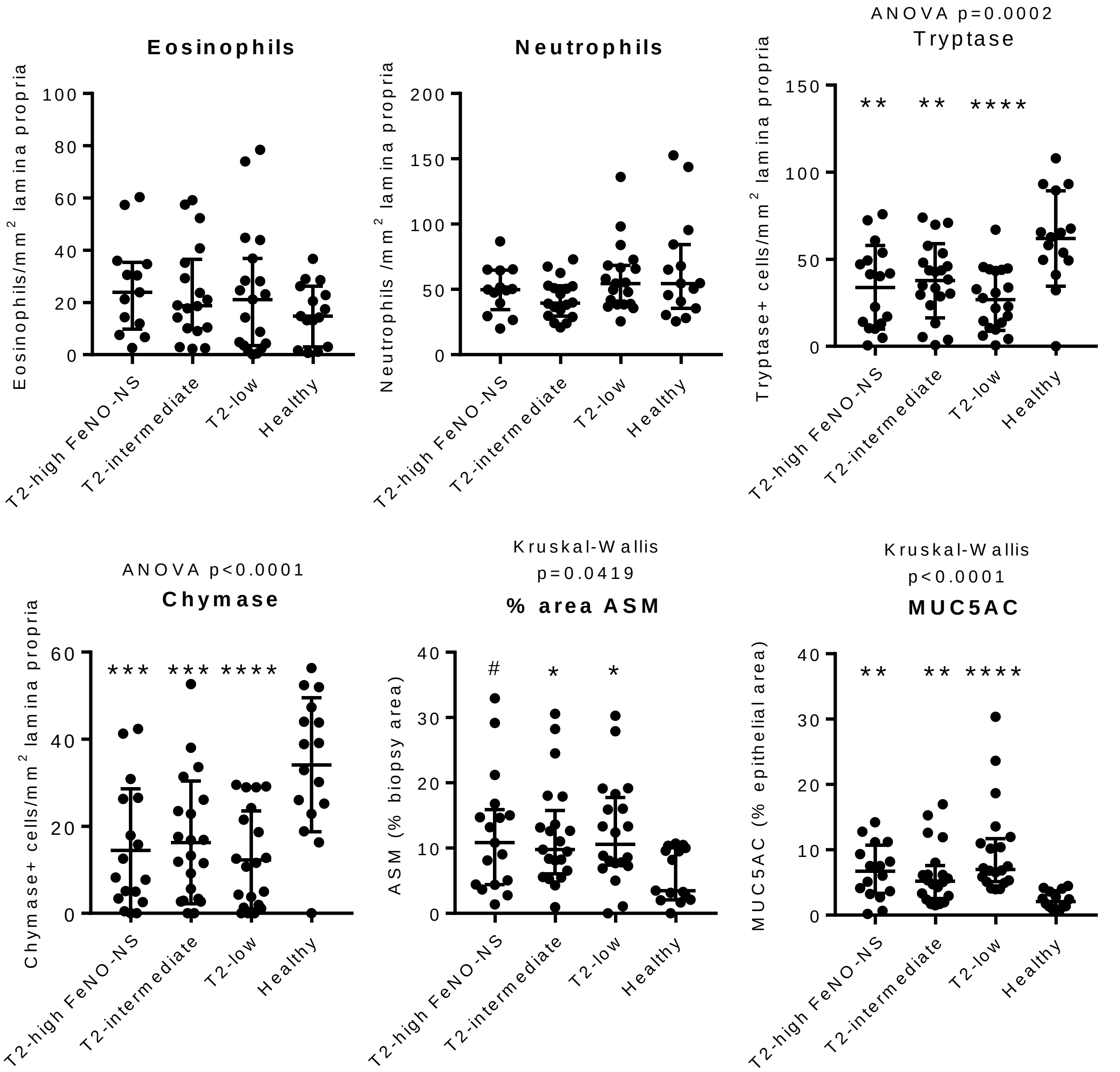

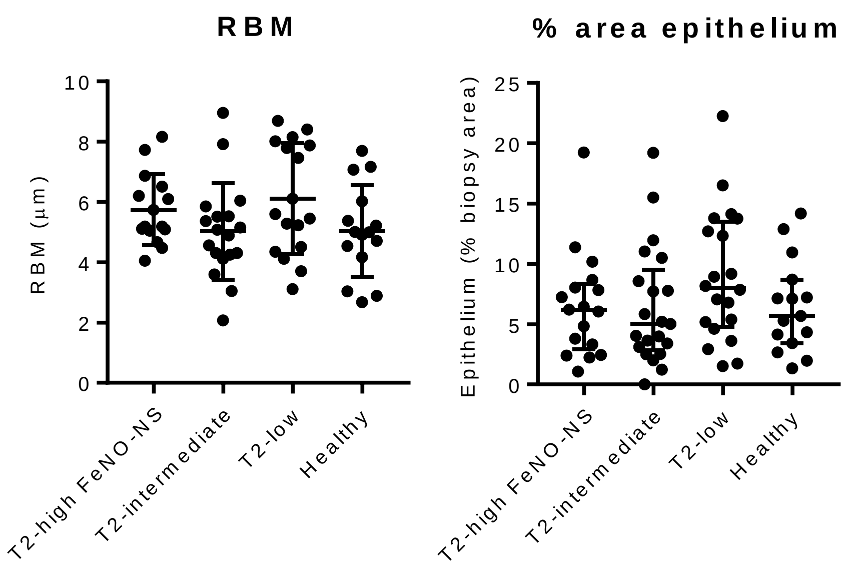

*p<0.05, **p<0.01, ***P<0.001, ****p<0.0001, #p=0.07 compared to healthy controls - Dunnett's or Dunn's multiple comparison tests as appropriate

Figure E8_Khalfaoui et al
