# Supplementary material for: Airway remodelling rather than cellular infiltration characterizes both type2 cytokine biomarker‐high and ‐low severe asthma
Source: Allergy. 2022 May 25;77(10):2974–86. doi: 10.1111/all.15376 (PMC9790286; doi:10.1111/all.15376)
Supplement: Supplementary file 9 — Figure S9 [file ALL-77-2974-s003.pdf]

Supplementary figure E9

Post hoc analysis with T2-low patients defined by blood eosinophils ≤ $0.15x10^9$/L and FeNO ≤25 ppb

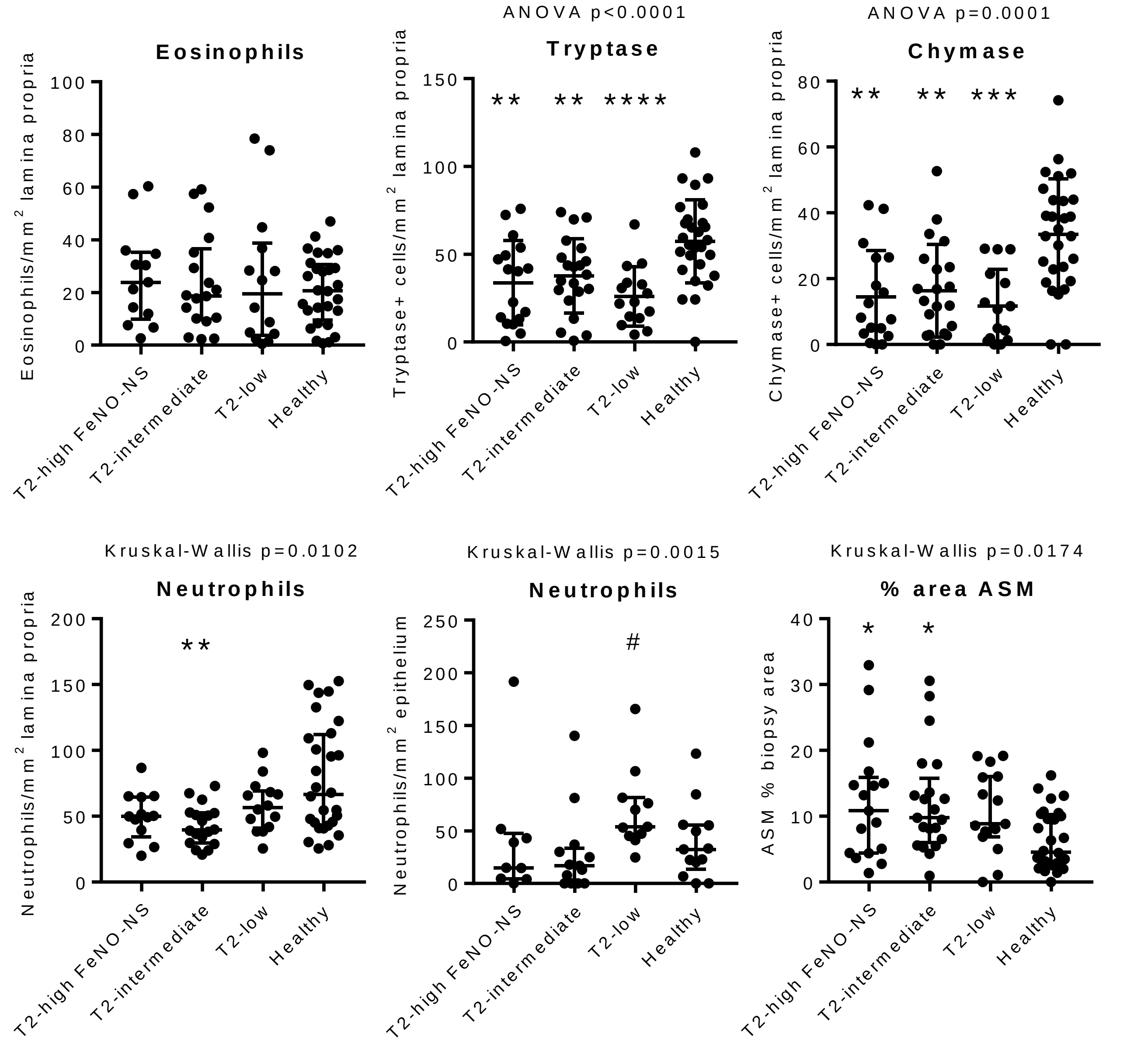

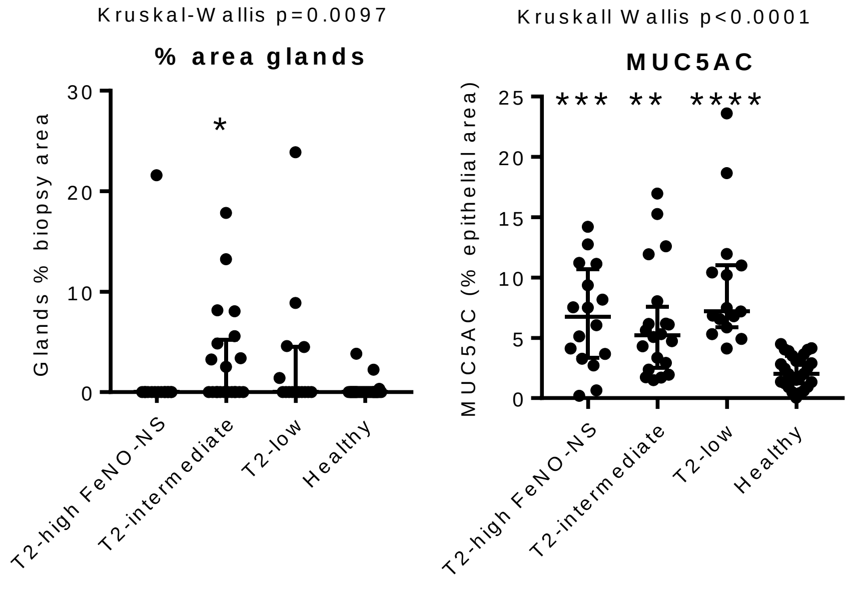

*p<0.05, **p<0.01, ***P<0.001, ****p<0.0001, #p=0.0068 compared to T2-intermediate - Dunnett's or Dunn's multiple comparison tests as appropriate.

For % ASM area, T2-high versus T2 low, p>0.9999.

Figure E9_Khalfaoui et al
