# Supplementary material for: Airway remodelling rather than cellular infiltration characterizes both type2 cytokine biomarker‐high and ‐low severe asthma
Source: Allergy. 2022 May 25;77(10):2974–86. doi: 10.1111/all.15376 (PMC9790286; doi:10.1111/all.15376)
Supplement: Supplementary file 10 — Appendix S1 [file ALL-77-2974-s001.docx]

**ONLINE SUPPLEMENT**

**Airway remodelling rather than cellular infiltration characterises both type2 cytokine biomarker-high and -low severe asthma**

^1^Latifa Chachi, PhD, ^1^Fiona A. Symon PhD, ^2^Simon Couillard MD, ^1^Beverley Hargadon RGN, ^3^Rekha Chaudhuri MD, ^3^Steve Bicknell FRCP,  ^4^Adel H. Mansur FRCP, PhD, ^2^Rahul Shrimanker MD, PhD, ^2^Timothy S.C. Hinks MD, PhD, ^2^Ian D. Pavord FMedSci, ^5^Stephen J. Fowler FRCP, MD, ^6^Vanessa Brown, PhD, ^6^Lorcan P. McGarvey FRCP, MD, ^6^Liam G. Heaney MRCP, MD, ^7^Cary D. Austin MD, PhD, ^8^Peter H. Howarth FRCP, DM, ^7^Joseph R. Arron MD, PhD, ^7^David Choy BSc, ^1^Peter Bradding FRCP, DM; on behalf of the UK Medical Research Council Refractory Asthma Stratification programme (RASP-UK)

^1^ Department of Respiratory Sciences, University of Leicester, Leicester Respiratory NIHR BRC, Glenfield Hospital, Leicester, UK.

^2^NIHR Oxford Respiratory BRC, Nuffield Department of Medicine, University of Oxford, Oxford, UK**.**

^3^ Gartnavel General Hospital, Glasgow, and Institute of Infection, Immunity and Inflammation, University of Glasgow, Glasgow, UK.

^4^University of Birmingham and Heartlands Hospital, University Hospitals Birmingham NHS Foundation Trust, Birmingham, UK.

^5^School of Biological Sciences, Faculty of Biology, Medicine and Health, University of Manchester, Manchester Academic Health Science Centre and NIHR Manchester Biomedical Research Centre, Manchester University Hospitals NHS Foundation Trust, Manchester, UK.

^6^ Centre for Experimental Medicine, Queen's University Belfast School of Medicine Dentistry and Biomedical Sciences, Belfast, UK.

^7^Genentech, Inc. South San Francisco, CA, USA.

# ^8^School of Clinical and Experimental Sciences, University of Southampton, NIHR Southampton Biomedical Research Centre, Southampton, UK

**Corresponding author**

Professor Peter Bradding, Department of Respiratory Sciences, University of Leicester, Glenfield Hospital, Leicester, LE3 9QP, UK. Tel: +44 116 258 3998

E-mail: [pb46@le.ac.uk](mailto:pb46@le.ac.uk)

**Funding sources**: This paper was supported by an MRC Stratified Medicine Grant MR/M016579/1, and in part by the National Institute for Health Research (NIHR) Leicester Biomedical Research Centre (Respiratory). Additional funding was received from Genentech to the University Hospitals of Leicester NHS Trust to support this study.

The views expressed are those of the author(s) and not necessarily those of the NHS, the NIHR or the Department of Health.

**METHODS**

The prospective study was approved by the East Midlands - Leicester South Research Ethics Committee (REC)(reference 16/EM/0260) and registered at clinicaltrials.gov (NCT02883530). Biopsy samples collected using the same standard operating procedure were also used from the pre-intervention arms of two other studies, i) a single centre bronchoscopy study to assess the effects of inhaled corticosteroids on adult healthy volunteers (referred to as Leicester HVS from here onwards) (NCT02476825, REC approval 15/EM/0313), and ii) a multi-centre study evaluating the effects of lebrikizumab on airway eosinophilic inflammation in participants with uncontrolled asthma^E1^ (referred to as Genentech CLAVIER from here onwards) (NCT02099656, independent ethics committee approval was obtained at all participating centres). All participants gave written informed consent.

**Patient populations**

**Inclusion criteria for people with severe asthma**

People with asthma met the following criteria for study entry:

1. Ability and willingness to provide written informed consent and to comply with the study protocol

2. Age 18–70 years at the time of informed consent

3. Severe asthma (BTS treatment step 4/5) despite intensive follow-up by an asthma specialist for at least 3 months

4. Diagnosis of asthma at least 12 months prior to informed consent

5. Baseline post bronchodilator FEV1 ≥ 40% of predicted

6. History of asthma treatment with high doses of inhaled glucocorticosteroids (≥ 1000 μg fluticasone propionate daily or equivalent (Clenil [BDP] 2000 μg, Fostair [BDP] 800 μg, fluticasone furoate 192 μg, budesonide dry powder 1600 μg, ciclesonide 640 μg), and LABA, with or without an additional controller, for at least 3 months prior to screening, or prior to corticosteroid optimisation as part of the RASP biomarker-guided study^E2^.

7. For patients using oral corticosteroids, adherence with their oral prednisolone regimen, as demonstrated by detectable serum prednisolone and evidence of suppressed cortisol within 6 hours of reported daily dose, on at least one occasion during the screening period or within the last 12 months is required.

8. Assessment according to the standards of the BTS UK Difficult Asthma Network or equivalent

9. Chest X-ray or computed tomography (CT) scan obtained within 12 months prior to consent (Visit 1) or chest X-ray during the screening period (prior to Visit 2) confirming the absence of other clinically significant lung disease

10. Documented history of bronchodilator reversibility response of ≥ 12% and ≥ 200 mL within the past 24 months, as demonstrated by any of the following:

i) Documented airflow obstruction (FEV_1_/forced vital capacity [FVC] <70%), where FEV_1_ has varied by ≥ 12% either spontaneously or in response to oral corticosteroid therapy, or

ii) Provocation challenge undertaken to local Trust policy which indicates the presence of positive airway hyperresponsiveness, or

iii) change in FEV_1_ by ≥ 12% and ≥ 200 mL after acute reversibility testing with 400 μg albuterol or 2.5–5 mg nebulized salbutamol

**Exclusion criteria for people with severe asthma**

People with asthma who met any of the following criteria were excluded from study entry:

- Treatment with intravenous [IV], intramuscular [IM]) or intraarticular corticosteroids within 4 weeks prior to Visit 1 or during the screening period for any reason, including an acute exacerbation event
- A severe asthma exacerbation requiring oral corticosteroids within 4 weeks, defined as an exacerbation of asthma requiring the new administration of oral steroids or an increase of at least 10 mg in their usual oral prednisolone dose within the last 4 weeks – defined from the last day of adjusted prednisolone therapy (such patients could re-screened >4 weeks from the last exacerbation)..
- Infection that meets any of the following criteria:
  - Any infection that resulted in hospital admission for ≥24 hours within 4 weeks prior to Visit 1 or during screening
  - Any infection that required treatment with IV or IM antibiotics within 4 weeks prior to Visit 1 or during screening
  - Any active infection that required treatment with oral antibiotics within 2 weeks prior to Visit 1 or during screening
  - Upper or lower respiratory tract infection within 4 weeks prior to Visit 1 or during screening
  - Antibiotics include any antimicrobial therapy used to treat bacterial, fungal, parasitic, viral, or other infections. Antibiotics prescribed for lung infection prophylaxis would also exclude the patient.
- Active tuberculosis requiring treatment within 12 months prior to Visit 1
  - Patients who have completed treatment for tuberculosis at least 12 months prior to Visit 1 and have no evidence of recurrent disease are permitted.
- Known immunodeficiency, including, but not limited to, HIV infection
- Evidence of acute or chronic hepatitis or known liver cirrhosis
- AST, ALT, or total bilirubin elevation ≥ 2.0 x the upper limit of normal (ULN) during screening
- Clinically significant abnormality on screening electrocardiogram (ECG) or laboratory tests (haematology, serum chemistry, and urinalysis) that, in the opinion of the investigator, may pose an additional risk of bronchoscopy
- History of clinically significant lung disease other than asthma
- Known current malignancy or current evaluation for a potential malignancy
- Unable to safely undergo elective flexible fiberoptic bronchoscopy because of any one of the following:
  - History of allergic reactions to local anesthetics to be used in the procedure
  - History of a clinically significant clotting abnormality, including on Screening Coagulation Panel
  - History of acute myocardial infarction, unstable angina, or other medical conditions that, in the opinion of the investigator, may make the patient unsuitable for elective bronchoscopy
- Other clinically significant medical disease that is uncontrolled despite treatment, that is likely, in the opinion of the investigator, to impact the patient's ability to participate in the study
- Current smoker, former smoker with smoking history of ≥15 pack-years
  - A current smoker was defined as someone who has smoked at least one cigarette per day (or pipe, cigar, or marijuana) for ≥ 30 days within the 12 months prior to Visit 1. A pack-year was defined as the average number of packs of cigarettes per day times the number of years of smoking.

• Use of a licensed or investigational monoclonal antibody including anti–IL 13, anti-IL-4/IL-13, omalizumab, anti–IL-5, or anti–IL 17, within 6 months or 5 drug half-lives prior to Visit 1 (whichever is longer) or during screening

• Use of a systemic immunomodulatory or immunosuppressive therapy (other than a monoclonal antibody or corticosteroids [see separate exclusion]) within 3 months or 5 drug half-lives prior to Visit 1 (whichever is longer) or during screening

• Use of other investigational therapy not described above within 4 weeks or 5 drug half-lives prior to Visit 1 (whichever is longer) or during screening

- Patients participating in a clinical trial that has not been unblinded should be assumed to have received the active drug

• Initiation of or change in allergen immunotherapy within 3 months prior to Visit 1 or during screening

• Receipt of a live attenuated vaccine within 4 weeks prior to Visit 1 or during screening

• Pregnant or lactating

• History of bronchial thermoplasty

**Inclusion criteria for healthy volunteers in the Leicester healthy volunteer study**

Healthy Subjects were required to have no respiratory co-morbidity or clinically important non-respiratory co-morbidity (including allergic rhinitis [perennial or seasonal], atopic dermatitis, food allergy). Participants consented to confirm that they provided accurate information regarding their medical/ surgical history and that a participants General Practitioner could be approached should further confirmation be required.

In addition, the following were required:

1. Participant was willing and able to give informed consent for participation in the study.

2. Male or Female, aged 18-65 years at Screening Visit 1

3. An FEV_1_ of >80% predicted

4. An FEV_1_/FVC ratio ≥70%

5. Non-smoker for at least 1 year with <10 pack year smoking history

6. Participant has clinically acceptable laboratory and ECG at screening (visit 1)

7. A chest x-ray (arranged at screening (visit 1)) that confirms the absence of clinically significant lung disease unless a chest X-ray or CT scan had been obtained within 12 months before screening (Visit 1) and is available for review by the investigator.

8. A negative result to skin prick testing with common aeroallergens (house dust mite, grass, tree-mix, cat, dog), or, if a skin prick test to a common aeroallergen is positive, a negative response to challenge with methacholine (defined as PC20 methacholine >16 mg/ml).

9. Able (in the Investigators opinion) and willing to comply with all study requirements.

10. Willing to allow his or her General Practitioner and consultant to be notified of participation in the study.

**Exclusion criteria** for the Leicester Healthy volunteer study were similar to those for the RASP study (see above).

**Inclusion criteria and exclusion criteria for the CLAVIER study** have been published previously^1^, and are similar to those for RASP. Tissue from nine participants with severe asthma who took part in Clavier and met the T2-low entry requirements for RASP were included in the analysis (this tissue was immunostained and analysed in Leicester along with the main study samples).

**Recruitment of people with severe asthma**

Participants underwent extensive evaluation at baseline including a full medical history, lung function testing, with bronchial challenge using methacholine where appropriate, and sputum inflammometry.

People with severe asthma were recruited prospectively from 3 sources: i) patients with a previous FeNO ≥45 ppb and blood eosinophils ≥0.3x10^9^/L, who had failed to suppress their FeNO during a FeNO suppression test^E3-5^ during routine clinical care, referred to as T2 biomarker-high FeNO-non suppressors (T2-high-FNS); ii) patients with a FeNO ≤30 ppb and blood eosinophils ≤0.2x10^9^/L identified in clinic, referred to as T2 biomarker-low (T2-low); this group was supplemented with 9 participants from the Genentech CLAVIER study^E1^, iii) patients who had exited the RASP T2-biomarker (FeNO, blood eosinophils, periostin)-driven treatment optimisation study^2^ with intermediate biomarker measurements (referred to as T2 biomarker-intermediate [T2-intermediate]) or low T2 biomarkers (as described for ii above). Current use of a biologic treatment was an exclusion criteria, and only one patient had used a biologic previously (omalizumab).

The rationale for the FeNO and blood eosinophil values defining group the T2-low group

was based on analysis of our existing cohorts of healthy subjects and patients with severe asthma in 2015 when the study was designed. The FeNO upper 95% CI of the mean was 29 ppb in our previous cohorts of healthy subjects, while the American Thoracic Society consider <25 ppb normal. The upper 95% CI of the geometric mean for blood eosinophils in our cohorts of healthy subjects was 0.16x10^9^/L, while the lower 95% CI for blood eosinophils in patients with a Th2-dependent gene signature was 0.31 x10^9^/L, and the lower 95% CI for blood eosinophils for those with a Th17 signature was 0.27 x10^9^/L. Based on these cut values and our prior definition of a Th2-high gene signature, we would be unlikely to identify Th2-high patients based on the proposed cut-offs in the T2-low group (Th2 and Th17 gene signature data from reference^E6^). A post hoc analysis using the more recent UK Severe Asthma Registry cuts for T2-low is also provided (blood eosinophils ≤0.15x109/L, FeNO ≤25 ppb).

Bronchoscopy

Subjects underwent bronchoscopy conducted according to British Thoracic Society guidelines^E7^. Mucosal biopsies were collected from 2^nd^-5^th^ generation bronchi under direct vision as per study procedure manual.

**Tissue processing and immunohistochemistry**

Biopsies were fixed in 4% neutral buffered formalin for 4 hours at 4°C as described for the Clavier study^E1^, then processed into paraffin wax, as per study procedure manual, with the same protocol used for all studies contributing to this analysis. Immunohistochemistry was performed in Leicester. All the laboratory procedures and processes were performed following the ISO9001-2015 Quality Management System and GCP/GLP guidelines.

3 µm tissue sections were cut and dewaxed, and stained with haematoxylin and eosin for quality control assessment, to ensure the integrity of the key tissue elements needed for immunohistochemistry staining and analysis. Further 3 µm sections were immunostained with the following mouse monoclonal primary antibodies: anti–neutrophil elastase (clone NP57, 0.42 μg/mL, Agilent Dako, UK), anti–mast cell tryptase clone AA1 (ready-to-use [RTU], Agilent Dako), anti-mast cell chymase (LS‑B12242, 0.8 μg/mL, LSBio, UK), anti–eosinophil major basic protein (clone BMK13, 1 μg/mL, Monosan, UK), anti-MUC5AC (RTU, Dako), anti-α-smooth muscle actin (αSMA)(RTU, Dako) and appropriate isotype controls (Dako and BD Biosciences). All immunostaining steps were performed using an Autostainer Link 48 (Agilent Dako, UK) using appropriate isotype controls followed by counterstaining in Gill’s haematoxylin. Two sections at least 18 μm apart were stained for each parameter.

**Assessment of immunopathology**

High-throughput morphologic analysis was performed on scanned sections using the Carl Zeiss Scanner Z1 and AxioCam HRc digital camera (Carl Zeiss, Germany). ZEN desk 3.1 image analysis software was used to perform image analysis on total, ASM, epithelium and lamina propria areas. The following previously validated pathological features^E8^ were evaluated as follows; i) nucleated inflammatory cells (eosinophils, neutrophils, mast cells [tryptase+ and chymase+]) were counted in the airway epithelium, lamina propria, ASM bundles, and airway glands, and expressed as cells/mm^2^ of the compartment of interest (ASM and epithelial area <0.05 mm^2^ were considered insufficient to quantify cell numbers); ii) the percentage of biopsy area occupied by epithelial, ASM and glandular structures was measured and expressed as a percentage of the total biopsy area; iii) MUC5AC expressed as the percentage of airway epithelium staining positive for MUC5AC was assessed using a thresholding technique as described previously^E9^; reticular basement membrane (RBM) thickness was expressed as the mean value of 50-points measurements approximately 20 µm apart according to the method validated by Sullivan et al^E10^. The mean of two sections at least 18 μm apart was taken for each immunohistological analysis.

All pathological data were assessed by an observer blinded to the identity of the patient.

**Sputum supernatant cytokine and lipid mediator measurements**

Induced sputum supernatants were collected in PBS. Cytokines (IL-4/-5/-13/-31/-33, CCL-17 (TARC), CCL-26 (eotaxin-3), TSLP, IFNγ, TNFα) were measured in duplicate using the Mesoscale Discovery platform according to the manufacturer’s instructions. Prostaglandin (PG)D_2_ and leukotriene (LT)E_4_ were measured by ELISA (Cayman Chemical, Michigan) according to the manufacturer’s instructions. For analysis the mean value of the duplicate measurements was used, and any samples below the limit of detection were assigned a value half the lowest limit of detection across the plates.

**Statistical analysis**

Basic summary statistical analysis was performed using GraphPad Prism version 7.03 (GraphPad Software, San Diego). Parametric and non-parametric data are presented as mean (standard error mean [SEM]) or median (interquartile range [IQR]) respectively.

For comparison of multiple groups characterised by continuous parametric and non-parametric variables the one-way analysis of variance (ANOVA) or Kruskal-Wallis test was used, followed by appropriate post hoc tests adjusted for multiple testing to explore within group differences (Sidak’s or Dunn’s multiple comparison tests). The χ^2^ test/Fisher’s exact tests were used to compare proportions. The Pearson correlation or Spearman’s rank correlation were used as appropriate to explore correlations between the data.

Due to the multiple groups and large number of permutations for multiple testing, two analyses were undertaken; one exploring differences between the asthma subgroups, the second comparing asthma overall versus health, with post hoc analysis comparing asthma phenotypes with health.

**RESULTS**

**The relationship between age and immunohistochemical parameters**

As the healthy control group was significantly younger than the asthma group, we explored the relationship between age and lamina propria and remodelling immunohistological parameters. The only relationship between age and the immunohistological parameters measured in the healthy control subjects was with lamina propria neutrophils (r_s_=-0.450, p=0.016 [p value unadjusted for measuring 9 lamina propria and remodelling immunohistological parameters]). This relationship was not present in people with asthma (rs=0.23, p=0.105), and there were no other relationships present in people with severe asthma. Re-analysing the neutrophil lamina propria data with healthy controls under the age of 25 removed to age-match the groups (leaving n=15, median age 50), removed the difference observed between asthma and healthy controls with regards to lamina propria neutrophil counts. The results remained unchanged for all other immunohistological parameters (**Figure E8**).

**The relationship between BMI and immunohistochemical parameters**

The healthy control group were leaner than those with asthma. The only relationship between BMI and inflammatory cell counts was with lamina propria tryptase+ mast cells in healthy controls (r_s_=0.503, p=0.006 [unadjusted for multiple comparisons]), and this was not present in people with asthma. There were no correlations in asthma or healthy controls between BMI and any remodelling feature.

REFERENCES

E1. Austin CD, Gonzalez Edick M, Ferrando RE, et al. A randomized, placebo-controlled trial evaluating effects of lebrikizumab on airway eosinophilic inflammation and remodelling in uncontrolled asthma (CLAVIER). *Clin Exp Allergy.* 2020;50(12):1342-1351.

E2. Heaney LG, Busby J, Hanratty CE, et al. Composite type-2 biomarker strategy versus a symptom-risk-based algorithm to adjust corticosteroid dose in patients with severe asthma: a multicentre, single-blind, parallel group, randomised controlled trial. *Lancet Respir Med.* 2020.

E3. Boddy CE, Naveed S, Craner M, Murphy AC, Siddiqui S, Bradding P. Clinical Outcomes in People with Difficult-to-Control Asthma Using Electronic Monitoring to Support Medication Adherence. *J Allergy Clin Immunol Pract.* 2020.

E4. Heaney LG, Busby J, Bradding P, et al. Remotely Monitored Therapy and Nitric Oxide Suppression Identifies Nonadherence in Severe Asthma. *Am J Respir Crit Care Med.* 2019;199(4):454-464.

E5. McNicholl DM, Stevenson M, McGarvey LP, Heaney LG. The utility of fractional exhaled nitric oxide suppression in the identification of nonadherence in difficult asthma. *Am J Respir Crit Care Med.* 2012;186(11):1102-1108.

E6. Choy DF, Hart KM, Borthwick LA, et al. TH2 and TH17 inflammatory pathways are reciprocally regulated in asthma. *Sci Transl Med.* 2015;7(301):301ra129.

E7. Du Rand IA, Blaikley J, Booton R, et al. British Thoracic Society guideline for diagnostic flexible bronchoscopy in adults: accredited by NICE. *Thorax.* 2013;68 Suppl 1:i1-i44.

E8. Siddiqui S, Shikotra A, Richardson M, et al. Airway pathological heterogeneity in asthma: Visualization of disease microclusters using topological data analysis. *J Allergy Clin Immunol.* 2018;142(5):1457-1468.

E9. Shikotra A, Choy DF, Ohri CM, et al. Increased expression of immunoreactive thymic stromal lymphopoietin in patients with severe asthma. *J Allergy Clin Immunol.* 2011;129:104-111.

E10. Sullivan P, Stephens D, Ansari T, Costello J, Jeffery P. Variation in the measurements of basement membrane thickness and inflammatory cell number in bronchial biopsies. *Eur Respir J.* 1998;12(4):811-815.

**SUPPLEMENTARY TABLE E1**

Baseline sputum demographics from the sputum mediator cohort

|  | T2-high FeNO-NS  [n=14] | T2-intermediate  [n=21] | T2-low  [n=8] | Healthy  (n=6) | p value† |
| --- | --- | --- | --- | --- | --- |
| Age - years | 59.0 (54.8-62.5) | 54.0 (46.5-68.0) | 48.0 (33.8-58.0) | 28.0 (22.3-61.0) | 0.0546 |
| Sex - M/F | 9/6 | 13/8 | 5/3 | 3/3 | 0.9437 |
| BMI (kg/m^2^) | 28.2 (24.6-36.8) | 31.4 (26.2-40.0)* | 30.2 (27.3-33.7) | 23.2 (20.9-27.2) | 0.0517 |
| Age onset - years | 48.0 (18.5-53.0) | 29.0 (6.0-48.5) | 11.5 (2.0-38.0) | N/A | 0.0691 |
| Ethnicity Caucasian - % | 100 | 90.5 | 100 | 100 | 0.4268 |
| Asthma duration - years | 14.0 (4.0-39.5) | 18.0 (11.5-40.0) | 30.0 (18.8-42.8) | N/A | 0.4184 |
| Atopic^Δ^ - % | 83.3 | 63.2 | 75.0 | 16.67 | **0.0411** |
| Annual exacerbation frequency | 1.5 (0.0-4.3) | 1.0 (0.0-2.0) | 3.5 (0.0-6.0) | N/A | 0.4339 |
| ICS dose – BDP equivalent - mcg | 2000 (2000-2000) | 2000 (2000-2000) | 2000 (2000-2000) | N/A | 0.9262 |
| Maintenance oral corticosteroids - % | 21.4 | 42.9 | 37.5 | N/A | 0.6668 |
| Ex smoker - % | 14.3 | 33.3 | 12.5 | 33.3 | 0.4660 |
| FEV_1_ Pre BD - L | 2.32 (1.94-2.69) | 2.43 (2.09-2.79) | 2.76 (2.07-3.30) | 3.38 (2.33-4.32) | 0.16678 |
| FEV_1_ Pre BD - % predicted | 67.5 (58.3-76.9)* | 78.2 (70.6-90.4) | 86.9 (62.9-98.2) | 95.4 (83.4-104.2) | **0.0233** |
| FEV1/FVC - % | 59.6 (55.9-68.6)*** | 66.8 (61,2-73.9)* | 66.9 (55.6-82.5) | 84.2 (80.4-89.3) | **0.0012** |
| ACQ5 | 1.1 (0.8-2.2) | 1.2 (0.6-2.1) | 1.9 (0.9-2.5) | N/A | 0.6547 |
| Total IgE – kU/L | 144 (108.5-274) | 110.0 (38-381) | 235.5 (73.8-410.3) | 31 (12.9-114.3) | 0.0844 |
| FeNO - ppb | 94.0 (48.8-119.3) **/ ##/§§§§ | 28.5 (17.3-37.7) | 15.0 (9.5-16.0) | 16.5 (11.5-27.5) | **<0.0001** |
| Blood eosinophils – x10^9^/L | 0.46 (0.25-0.64)§§ | 0.24 (0.10-0.31) | 0.09 (0.02-0.22) | 0.14 (0.09-0.26) | **0.0064** |
| Sputum eosinophils (%) | 11.0 (3.0-31.5)** [n=11] | 1.5 (0.1-9.0) [n=19] | 0.3 (0.0-0.3)[n=7] | 0.0 (0.0-0.1)[n=5] | **0.0038** |
| Sputum neutrophils (%) | 28.8 (2.2-67.3)  [n=11] | 32.5 (3.0-88.0)[n=19] | 66.5 (21.0-85.0)[n=7] | 53.2 (6.5-94.0)[n=5] | 0.4859 |

Continuous variables are presented as mean ± SD or median (interquartile range). BMI = Body Mass Index. BD =Bronchodilator, BDR = Bronchodilator Reversibility. All tests for continuous variables are ANOVA or Kruskal Wallis across all groups unless indicated otherwise, with adjusted p-values for between group comparisons obtained using Sidak’s or Dunn’s multiple comparison tests. For categorical variables, a Chi-Squared test was used across applicable groups.

*P<0.05, **p<0.01, ***p<0.001, ****p<0.0001 compared to healthy control subjects. #p<0.05, ##p<0.01 compared to T2-intermediate. §p<0.05, §§p<0.01, §§§§p<0.0001 compared to T2-low.

Please note: Sputum supernatants were available from 34/54 of the bronchoscopy cohort described in the main text. Sputum supernatants were also available from 2 people without bronchoscopic samples collected for immunohistochemistry, and 7 participants who passed screening but did not proceed to bronchoscopy, commonly because they withdrew or exacerbated. The clinical characteristics of these 43 patients and 6 healthy controls summarised in table E1 above are therefore different to those in main paper.

^Δ^Atopy refers to the presence of a positive skin test or the presence of a raised specific IgE to a common aeroallergen
