# Supplementary material for: Airway remodelling rather than cellular infiltration characterizes both type2 cytokine biomarker‐high and ‐low severe asthma
Source: Allergy. 2022 May 25;77(10):2974–86. doi: 10.1111/all.15376 (PMC9790286; doi:10.1111/all.15376)
Supplement: Supplementary file 11 — Appendix S2 [file ALL-77-2974-s010.docx]

**SUPPLEMENTARY TABLE E1**

Baseline sputum demographics from the sputum mediator cohort

|  | T2-high FeNO-NS  [n=14] | T2-intermediate  [n=21] | T2-low  [n=8] | Healthy  (n=6) | p value† |
| --- | --- | --- | --- | --- | --- |
| Age - years | 59.0 (54.8-62.5) | 54.0 (46.5-68.0) | 48.0 (33.8-58.0) | 28.0 (22.3-61.0) | 0.0546 |
| Sex - M/F | 9/6 | 13/8 | 5/3 | 3/3 | 0.9437 |
| BMI (kg/m^2^) | 28.2 (24.6-36.8) | 31.4 (26.2-40.0)* | 30.2 (27.3-33.7) | 23.2 (20.9-27.2) | 0.0517 |
| Age onset - years | 48.0 (18.5-53.0) | 29.0 (6.0-48.5) | 11.5 (2.0-38.0) | N/A | 0.0691 |
| Ethnicity Caucasian - % | 100 | 90.5 | 100 | 100 | 0.4268 |
| Asthma duration - years | 14.0 (4.0-39.5) | 18.0 (11.5-40.0) | 30.0 (18.8-42.8) | N/A | 0.4184 |
| Atopic^Δ^ - % | 83.3 | 63.2 | 75.0 | 16.67 | **0.0411** |
| Annual exacerbation frequency | 1.5 (0.0-4.3) | 1.0 (0.0-2.0) | 3.5 (0.0-6.0) | N/A | 0.4339 |
| ICS dose – BDP equivalent - mcg | 2000 (2000-2000) | 2000 (2000-2000) | 2000 (2000-2000) | N/A | 0.9262 |
| Maintenance oral corticosteroids - % | 21.4 | 42.9 | 37.5 | N/A | 0.6668 |
| Ex smoker - % | 14.3 | 33.3 | 12.5 | 33.3 | 0.4660 |
| FEV_1_ Pre BD - L | 2.32 (1.94-2.69) | 2.43 (2.09-2.79) | 2.76 (2.07-3.30) | 3.38 (2.33-4.32) | 0.16678 |
| FEV_1_ Pre BD - % predicted | 67.5 (58.3-76.9)* | 78.2 (70.6-90.4) | 86.9 (62.9-98.2) | 95.4 (83.4-104.2) | **0.0233** |
| FEV1/FVC - % | 59.6 (55.9-68.6)*** | 66.8 (61,2-73.9)* | 66.9 (55.6-82.5) | 84.2 (80.4-89.3) | **0.0012** |
| ACQ5 | 1.1 (0.8-2.2) | 1.2 (0.6-2.1) | 1.9 (0.9-2.5) | N/A | 0.6547 |
| Total IgE – kU/L | 144 (108.5-274) | 110.0 (38-381) | 235.5 (73.8-410.3) | 31 (12.9-114.3) | 0.0844 |
| FeNO - ppb | 94.0 (48.8-119.3) **/ ##/§§§§ | 28.5 (17.3-37.7) | 15.0 (9.5-16.0) | 16.5 (11.5-27.5) | **<0.0001** |
| Blood eosinophils – x10^9^/L | 0.46 (0.25-0.64)§§ | 0.24 (0.10-0.31) | 0.09 (0.02-0.22) | 0.14 (0.09-0.26) | **0.0064** |
| Sputum eosinophils (%) | 11.0 (3.0-31.5)** [n=11] | 1.5 (0.1-9.0) [n=19] | 0.3 (0.0-0.3)[n=7] | 0.0 (0.0-0.1)[n=5] | **0.0038** |
| Sputum neutrophils (%) | 28.8 (2.2-67.3)  [n=11] | 32.5 (3.0-88.0)[n=19] | 66.5 (21.0-85.0)[n=7] | 53.2 (6.5-94.0)[n=5] | 0.4859 |

Continuous variables are presented as mean ± SD or median (interquartile range). BMI = Body Mass Index. BD =Bronchodilator, BDR = Bronchodilator Reversibility. All tests for continuous variables are ANOVA or Kruskal Wallis across all groups unless indicated otherwise, with adjusted p-values for between group comparisons obtained using Sidak’s or Dunn’s multiple comparison tests. For categorical variables, a Chi-Squared test was used across applicable groups.

*P<0.05, **p<0.01, ***p<0.001, ****p<0.0001 compared to healthy control subjects. #p<0.05, ##p<0.01 compared to T2-intermediate. §p<0.05, §§p<0.01, §§§§p<0.0001 compared to T2-low.

Please note: Sputum supernatants were available from 34/54 of the bronchoscopy cohort described in the main text. Sputum supernatants were also available from 2 people without bronchoscopic samples collected for immunohistochemistry, and 7 participants who passed screening but did not proceed to bronchoscopy, commonly because they withdrew or exacerbated. The clinical characteristics of these 43 patients and 6 healthy controls summarised in table E1 above are therefore different to those in main paper.

^Δ^Atopy refers to the presence of a positive skin test or the presence of a raised specific IgE to a common aeroallergen
